# Modeling Long-Term Dynamics of Biogenic Volatile Organic Compounds (BVOCs) in Germany Based on Major Precursors

**DOI:** 10.1021/acs.est.4c14418

**Published:** 2025-02-26

**Authors:** Ayoub Moradi, Temesgen Alemayehu Abera, Elliot Samuel Shayle, Mohammed Ahmed Muhammed, Dirk Zeuss

**Affiliations:** Department of Environmental Informatics, Faculty of Geography, Philipps University of Marburg, Marburg 35032, Germany

**Keywords:** air quality, climate change, CO_2_, remote sensing, biomass, vegetation index

## Abstract

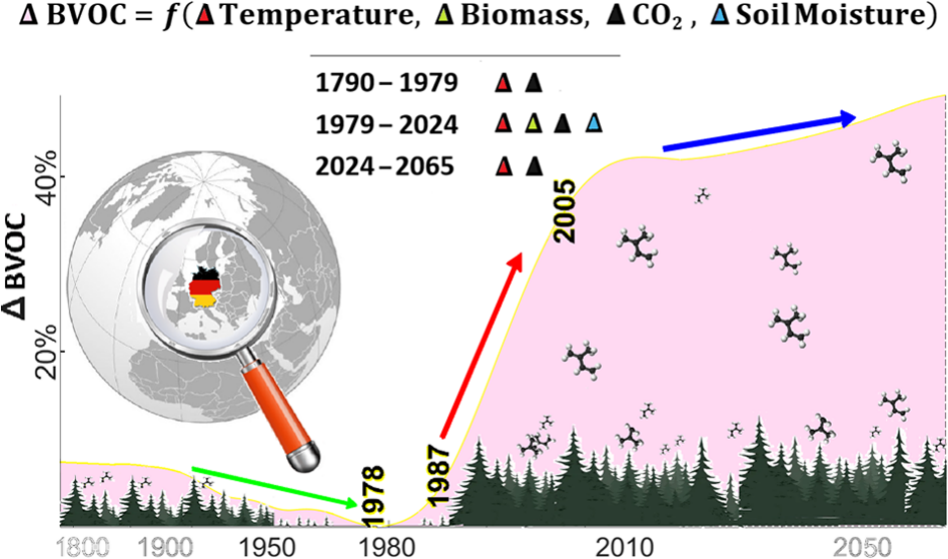

Biogenic Volatile Organic Compounds (BVOCs) are organic chemicals emitted primarily by flora. They impact climate and human health, and their presence is influenced by environmental conditions. Despite the intense effects of climate change, there is lack of information about the spatiotemporal variations in BVOC emissions and underlying driving factors. This study aims to quantify the variation of BVOCs in Germany and attribute it to deriving factors. We first conducted a detailed study covering the period from 1979 to 2024, during which satellite observations were first became available. Then, using historical records and projected future climate-change scenarios, we simulated the dynamics of BVOCs over an extended timeframe (1790–2065). To track changes in BVOCs emissions, we accounted for constant emission factors and used remotely sensed proxies, summarized into five main factors: biomass, temperature, carbon dioxide, soil moisture, and wildfire. Our results show that the trend of BVOCs emissions declining reversed in 1978 ± 1, with increases now being observed. Since this year, the average BVOCs emissions in Germany increased by ∼54%. Spatially, eastern-central Germany and urban areas exhibit the highest increase. Future projections indicate a continued increase in emissions over the coming decades. Our results were validated by analyzing the Total Column Ozone and Formaldehyde.

## Abstract 2

Since 1978, air quality in Germany has shown notable changes, with biogenic volatile organic compounds increasing by over 50% after reaching their lowest levels post-industrial revolution.

## 1 Introduction

Biogenic Volatile Organic Compounds (BVOCs) are a class of organic compounds emitted by living organisms as they interact with the atmosphere,^1^ although VOCs are also influenced by urban activities.^2−5^ BVOCs significantly impact the chemistry of the atmospheric, ecology, and climate^6−10^ and can contribute to the formation of ground-level ozone, an airborne pollutant which is harmful to humans.^11^ Following oxidation in the atmosphere, BVOCs form Secondary Organic Aerosols (SOAs),^12^ which additionally affects air quality and climate by scattering sunlight^13^ and acting as cloud condensation nuclei.^14,15^ Moreover, BVOCs interact with greenhouse gases such as methane, thus contributing to climate change.^16^ BVOCs have a wide range of chemicals. In most ecosystems, however, isoprenes, monoterpenes, and sesquiterpenes together constitutes ∼80% of BVOCs.^17,18^ Plants are the primary source of BVOCs, emitting them as a byproduct of photosynthesis, and as responses to environmental stressors.^19^ The genetic structure of plant plays a crucial role in determining the types and amounts of BVOCs they emit.^20^ Plants may emit BVOCs defensively as a response to abiotic stressors, e.g., extreme temperatures, drought, and UV-radiation;^21−23^ and biotic stressors, e.g., herbivory and pathogen attack.^24,25^ The metabolic processes of bacteria and other soil-dwelling microbes also contribute to BVOCs emission.^19^ Lastly, wildfires release large amounts of BVOCs into the atmosphere.^26^

The Model of Emissions of Gases and Aerosols from Nature (MEGAN) is a widely used tool for modeling BVOCs emissions. MEGAN simulates the emission of BVOCs based on plant type and environmental conditions.^27−29^ Different models also exist to provide a variety of insights. These include: GUESS-ES (Lund-Potsdam-Jena General Ecosystem Simulator with Emission Module);^30^ EMEPMSC-W (European Monitoring and Evaluation Program Meteorological Synthesizing Centre-West);^31^ and ORCHIDEE (Organizing Carbon and Hydrology in Dynamic Ecosystems).^32^ BVOCs emissions have traditionally been measured at small scales using ground-based tools such as eddy-covariance and mass spectrometry.^33^ MEGAN and similar models typically rely on detailed land cover and environmental factors, which are often limited in temporal and spatial resolution. While these models are useful for estimating emissions and capturing short-term fluctuations, their application in anomaly analyses, such as in our study, introduces unnecessary complexity to the workflow and may compromise the accuracy of the estimated anomalies.

Emerging remote sensing technologies offer a promising alternative for modeling and in situ measuring. They can capture signatures on land cover and environmental conditions, which are two key indicators of BVOCs emissions. These spectral attributed criteria, known as Vegetation Indices (VI), are powerful tools to be able to quantify the extent, density, and composition of vegetation cover.^33,34^ The number of scientific papers which report on BVOCs has increased considerably during the previous two decades.^35^ Wang et al.^36^ showed that BVOCs emissions worsened air quality in China. Significant regional and global trends in BVOCs emissions have been identified through several studies.^37^ Research conducted in Hamburg in northern Germany assessed the effect of abiotic stressors on BVOCs emissions from urban green infrastructure.^38^ Global warming has affected Germany, leading to a warmer climate and increased greenness.^39^ Additionally, the CO_2_ concentration highly varied after the industrial revolution. Due to the extensive vegetation coverage, variable atmospheric CO_2_, and significant impact of global warming, Germany represents an exemplary study area to investigate the dynamics of BVOCs emissions. Despite climate change’s impact over recent decades, comprehensive interannual variations and spatial pattern of BVOCs emissions in Germany are not yet well understood. Understanding the spatiotemporal distribution of emissions is essential for developing atmospheric models and implementing effective environmental policies to mitigate or reduce the ongoing negative impacts of the global climate changes on air quality. Furthermore, Germany’s commitment to environmental preservation and restoration necessitates research that can address climate change. This study aims to quantify variation in BVOCs emissions by assessing the effects of changes in biomass and environmental factors on the emissions. Biomass is modeled through established relationships between field-recorded samples and corresponding remotely sensed VIs.^40−42^ We, moreover, explore various methodologies and applications of the remote sensing data sets, highlighting the advantages and challenges associated with each.

The article is structured in five sections: after this introduction, material and methodologies are presented which include brief definition on the study area, BVOC metrics and influencing factors, and biomass estimation, with details addressed in the Supporting Information. Then, in Section 3, the Result Section includes estimation of each contributor to BVOC, seasonality analysis, spatial analysis, and past and future perspectives. Section 4 is allocated to evaluation and assessment of results. And, the Discussion is brought to Section 5.

## 2 Materials and Methods

### 2.1 Study Area

Germany, located in Central Europe, encompasses a variety of ecosystems, ranging from the lowlands to the Alpine regions. Its extensive vegetation coverage makes it an ideal location for studying BVOCs. Forest and agricultural lands, the largest sources of BVOC emissions, cover about one-third and 50% of the country, respectively. Further details on the study area are in Text S1.

### 2.2 BVOCs Emissions Calculation

In a given time and place, BVOCs emissions are calculated by multiplication of Emitter and Emission-Factor (EF).^27,28^ Emitter is the unit of the plant area or biomass, and EF is a specific coefficient that quantifies emissions per unit of the emitter. EF consists of a constant species-specific factor and a variable scaling factor (SF) that accounts for environmental conditions
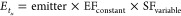


For our study, which does not focus on estimation of BVOC emissions rates, we simplify the estimation of changes by eliminating the constant species-specific EF, resulting in
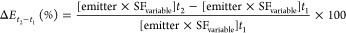
Here, variation in the “Emitter” is attributed to changes in vegetation cover, such as shifts between different vegetation types or between vegetated and nonvegetated areas.

The continuously changing Scaling Factor (SF) can be decomposed into several components
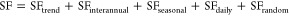


We focused on trend and interannual signals, while any periodic (e.g., seasonal biomass fluctuations) or daily fluctuations are generally stationary over decades. However, we also analyzed potential variations in seasonality over the study period

### 2.3 Influencing Factors on BVOCs Emissions

The emission rates of BVOCs are influenced by various factors, which can be broadly categorized into biological and environmental factors. Considering data availability, we analyzed 11 factors, including shortwave radiation (SWR), temperature (T), vegetation indices including: Normalized Difference Vegetation Index (NDVI), Leaf Area Index (LAI), and Fraction of Absorbed Photosynthetically Active Radiation (FAPAR), precipitation (P), soil moisture (SM), cloud cover (CC), carbon dioxide (CO_2_), wildfire frequency (WF-N), and wildfire burnt area (WF-A). Each factor is discussed individually in Text S2. Normalized trends (Figure S8) indicate that SWR and CO_2_ have the highest increases and decrease, respectively. The increase in SWR has resulted in higher temperatures and enhanced vegetation development, as reflected in the vegetation indices. Among the factors, only the decreasing trend of soil moisture leads to a reduction in BVOCs emissions. Rainfall may have varying impacts depending on other environmental conditions; however, its effect on BVOCs emissions is minimal due to its negligible trend. The examined factors provide a comprehensive understanding of the reasons behind long-term variations in BVOCs emissions. Nevertheless, these factors are not entirely independent, so their effects cannot be simply accumulated to estimate BVOCs. Consequently, the factors were summarized into five categories with maximum independence: vegetation live biomass, temperature, soil moisture, CO_2_, and wildfire burnt biomass (Figure S9). These categories have distinct effects on BVOCs emissions, which may be measured independently. Unlike the initial ranking (Figure S8), in the ranking of independent factors, the effect of vegetation (biomass) is the most significant.

### 2.4 Vegetation Types as the Base Land Cover

Environmental and ecological models use Plant Functional Types (PFTs) to parametrize vegetation-based processes.^43,44^ To estimate BVOC emissions, we used PFTs as standard vegetation inputs, following models like MEGAN. The European Space Agency’s Climate Change Initiative provides a high-resolution (300 m) PFT time series data set from 1992 to 2020.^45^ Direct mapping of PFTs is challenging, but spectral data can indirectly indicate the PFT variables. For the years 2021 to 2024, we generated PFT maps from 500m resolution MODIS data using the Random Forest algorithm (Figure S1). For 1981 to 1992, we used AVHRR-NOAA data based on a constant vegetation map from PFT–1991. Statistical analysis of the ESA’s 29–year PFT time series indicates that the target classes in Germany have remained largely stable, with ∼95% of pixels showing no significant change (Table S1).

### 2.5 Biomass Estimation

Biomass refers to the total mass of living plants in a given area. Two main methods for estimating BVOC emissions are biomass-based and vegetation index (VI)- based^1,7,46^ approaches. Biomass data, which include species-specific information, allow for more precise estimations, but existing biomass measurements are often only available annually and may miss seasonal variations. In the other hand, most models are developed to identify the spatial distribution of biomass at a specific time, often when vegetation biomass is at its peak. Consequently, these models are not suitable for differentiating biomass over time. Conversely, VIs like the NDVI and LAI, providing information about vegetation extent and canopy volume, respectively, can capture seasonal variations in leaf area but lack species-specific detail. Biomass models can be species-specific and region-specific; thus, different species with similar VI values may have considerably different biomass and thus different BVOCs emissions rates. This is particularly important for agricultural areas where crop biomass may proportionally differ, noting that agricultural farms cover more than 50% of the Germany total area. For large-scale estimations, VI-based estimation is more practical. To address this, we combined both approaches to maximize the capture of BVOCs emissions variations. Remote sensing has made it possible to model biomass from spectral indices and limited ground measurements.^47,48^ NDVI and LAI are commonly used to estimate biomass in various types of vegetation via regression modeling.^49,50^ When choosing between NDVI and LAI, we considered two criteria: the rate of change and the separability between vegetation classes (Figure S2). NDVI was selected for biomass estimation based on its performance across different vegetation types. Details of the biomass models are presented in Text S3. We processed remotely sensed vegetation indices and environmental data using the Google Earth Engine platform.^51^

## 3 Results

### 3.1 Estimation of BVOCs Emissions Anomaly: 1979–2024

#### 3.1.1 Biomass Contribution

the impact of biomass on BVOCs emissions is straightforward. Empirical measurements have demonstrated a positive proportional relationship between biomass and the BVOCs emissions.^52^ We calculated the biomass trends in forest (f), agriculture (a) and grasslands (g) according to NDVI trends (Figure S2), and NDVI-biomass regression models (Figure S4). The area-weighted biomasses have resulted in a total 24% increase in BVOC emissions, with subcontributions of 8.34%, 14.05%, and 1.65% from forest, agriculture, and grasslands, respectively.
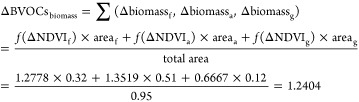


#### 3.1.2 Temperature Contribution

BVOC emissions typically increase with rising temperatures, following a simplified exponential relationship known as the Q10 for temperatures below 30 °C, where the emission rate approximately doubles with every 10 °C increase in temperature.^1,27,53^ A more precise model for estimating Temperature Correction Factors (γ*T*) uses a constant coefficient of β, often set at 0.1. The MEGAN model, however, uses species-specific γ values, which generally range between 0.1 and 0.2. Based on the observed temperature trend (1.88 °C), BVOC emissions have increased by 20.7%.
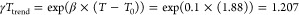


#### 3.1.3 CO_2_ Contribution

CO_2_-induced BVOC variation is generally referred to isoprene emission; and there is no statistically significant effect of CO_2_ concentration on the other components.^54,55^ The MEGAN similarly does not consider the CO_2_ level for components other than isoprene. The adjustment for isoprene emission can be estimated as follow
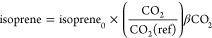


where βCO_2_ is the CO_2_ sensitivity factor derived from experimental data, which varies depending on the genotype of the trees. The MEGAN applies an average sensitivity factor of 50%. Needle-leaved trees typically exhibit a lower CO_2_ sensitivity compared to broadleaved trees. In Germany, approximately 54% of forests are composed of coniferous trees, which have a sensitivity factor ranging from 25 to 30%. For this analysis, we assumed an average sensitivity factor of 33%, which resulted in a 17% increase in isoprene for a 38% decrease in CO_2_. Regarding that isoprene constitutes about half of the total BVOC, the CO_2_ induced variation in total BVOC is estimated at 8.5%
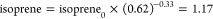

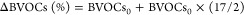


#### 3.1.4 Soil Moisture Contribution

Long-term impact of soil moisture variations on plant BVOC emission is reflected in biomass variations. However, the soil moisture change may also influence the emission from soil bacteria. Research generally indicates that increased soil moisture leads to a higher production of BVOCs from soil. Although, the impact varies across studies, a linear model is sufficient to explain BVOC flux from soil.^56^ The contribution of soil moisture is estimated linearly as its long-term anomaly (see Figure S6b), giving a 5.28% decrease in the BVOC emissions
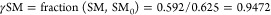
where, SM and SM_0_ are current and reference soil moistures, respectively (see Figure S6b).

#### 3.1.5 Wildfire Contribution

The BVOCs emissions from burnt biomass are typically calculated based on the amount of dry biomass, which is scaled from the green biomass using a recommended scaling factor of 0.5^57,58^ in the literature. The largest wildfire in recent decades occurred in 1996′s in Lüneburg Heath, burning an area of 4840 ha. The dominant tree species in Lüneburg, Scots Pine, and English Oak, have the emission factors of 0.5 and 0.2 g/kg of dry burnt biomass, respectively.^59,60^ Assuming that 90% of the burnt area was forest, and taking 293 ton/hectare as the biomass of temperate forest, 0.638 million tons of dry biomass has been burnt, leading to 223.3 tons of total emission during the Lüneburg Heath’s wildfire
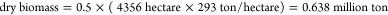

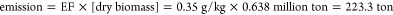


Compared to the typical emission rate of approximately 0.26 kg/hectare/day in a temperate forest,^27,61^ the estimated emission from the Lüneburg Heath wildfire (∼2.4 kg/hectare/day) is 9.3 times higher. However, since wildfires are discrete sources of BVOC emissions, their impact on weekly and monthly scales can be significant.

#### 3.1.6 Additional Contribution Due to Added Biomass

The impact of environmental factors on the increased biomass are accounted for as follows
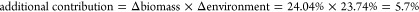


Net BVOCs Emissions is the sum of the contributions
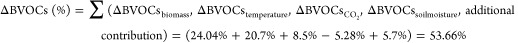


**Figure 1:**
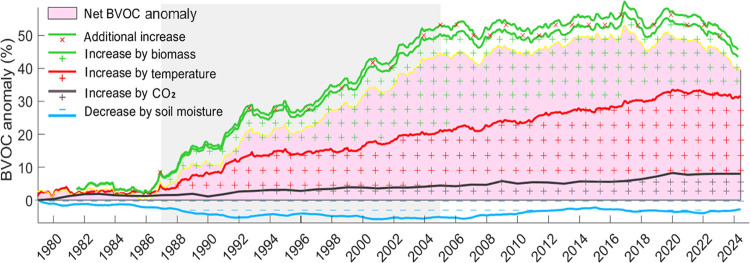
Long-term variations in BVOC emissions, smoothed using 7-year moving averages. The gray-shaded area indicates the period of the highest increase.

Although the data processing resulted in a unified resolution, the data sets used varied significantly in their time resolution, ranging from 8-day VIs to yearly biomass measurements. Consequently, the final estimates are influenced by this range. Additionally, changes in vegetation indices do not always correspond immediately to biomass changes and, by extension, to BVOC emissions. Lag effects arise from factors, such as seasonal growth patterns, phenological changes, and the time required for plants to respond to environmental conditions. To reduce temporal inconsistency and eliminate periodic fluctuations, we smoothed the estimates over 4-year and 7-year intervals (Figure 1). The most significant increase, with a slope of 2.4%/year, occurred between 1987 and 2005 due to a rapid rise in biomass. From 2006 to 2020, this increase slowed to about 0.6%/year, and post-2020, it showed a decrease of 1.2%/year. The interannual variations were primarily influenced by biomass changes.

### 3.2 Seasonality Analysis: 1979–2024

BVOCs emissions generally exhibit clear seasonal patterns, with higher rates in the summer and lower rates in the winter. These patterns are primarily influenced by fluctuations in the temperature and biomass. Analysis of temperature time series indicated that over the studied period, minimum and maximum air temperatures increased by 1.65 and 2.15 °C, respectively (Figure S10), resulting in a 0.5 °C increase in seasonal temperature fluctuation. According to the Q10 effect, this temperature change could amplify seasonal BVOCs emissions by 5.13%. Additionally, the analysis of vegetation indices reveals increased seasonal amplitudes for NDVI and LAI (Figure S11). In contrast, FAPAR does not show a significant increase as it is influenced more by vegetation structure, which has remained stable. Although there should have been an increase in seasonal biomass fluctuation, this cannot be accurately estimated due to the lack of interseasonal biomass sampling. Consequently, we adopted the NDVI-biomass models to infer the seasonal biomass increase proportional to the trend contributions of NDVI to biomass increase. Therefore, based on NDVI-Biomass relationships (Figure S4), the increase in seasonal NDVI (9.5%) could have increased seasonal biomass and thus the seasonal BVOCs emissions by 6.13%, resulting in a total seasonal increase of 11.26%. No significant change in the seasonal amplitude of soil moisture was observed. It should be declared that the estimated seasonal increase is an average; thus, it may slightly vary in different vegetation types.

### 3.3 Spatial Analysis: 1979–2024

**Figure 2:**
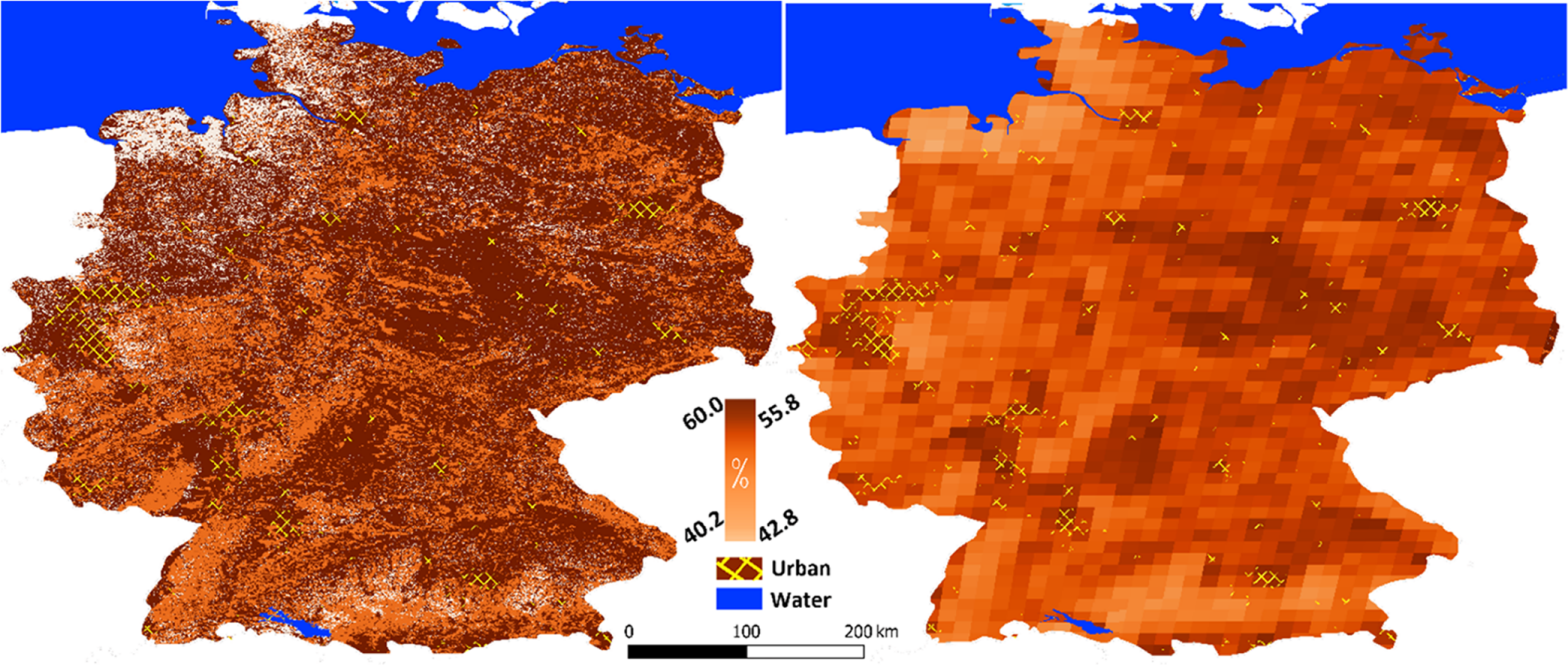
Spatial distribution of BVOCs emissions changes (%) from 1979 to 2024 in Germany, based on original 500m MODIS pixels size (left) and smoothed over 15km (right).

Globally, the distribution of BVOC emissions closely matches that of vegetation type maps. However, changes in emission rates can vary significantly from region to region due to climate change and anthropogenic impacts. Trends in temperature, soil moisture, and CO_2_ are highly homogeneous across Germany, however biomass variation is considerably heterogeneous which is the base for spatial distribution of the anomaly in the BVOC emission. The greatest increase in emissions is observed in the eastern-central part of Germany, where biomass (NDVI) has experienced a higher increase due to development of agriculture. Conversely, the smallest increase is found in the northwestern coastal regions along the North Sea, where natural grasslands dominate (Figure 2, left). Generally, urban areas show the highest increases (>50%), largely due to their proximity to agricultural lands and the expansion of green spaces within the cities. BVOCs typically propagate through the atmosphere from their sources, influenced by meteorological conditions such as wind speed and vertical mixing. Particularly, isoprene and longer-lived secondary products, e.g., SOA a ground-level ozone, may propagate over several kilometers.^62,63^ To account for spatial propagation, we use an average distance of 15 km for the transfer and mixing of BVOCs (Figure 2, right).

### 3.4 Past and Future Perspectives

Data on vegetation dynamics prior to the advent of satellite observations are sparse, limited to small-scale records. However, regional vegetation conditions can be inferred with reasonable accuracy using primary climatological factors which have been available from the Global Land Data Assimilation System (GLDAS version 2)^64^ since 1948. The GLDAS is a sophisticated data system that integrates satellite and ground-based observations with advanced land surface models to deliver estimates of various land surface conditions worldwide. For estimating BVOCs emissions, temperature is a crucial factor, as it directly influences emission rates and indirectly affects emissions through its impact on plant development. We also analyzed the time series of precipitation. While average precipitation varies between forested and agricultural areas, it does not exhibit a long-term trend (Figure S12). In contrast, the temperature shows a clear and intensifying trend over the two specific epochs relevant to our study (Figure S13). Comparisons reveal that warming in forest areas has accelerated, surpassing the temperature increases observed in agricultural regions. This difference may be attributed to the cooling effects of agricultural development post-1978, which have led to an increased contribution of latent heat. We adjusted biomass and BVOCs emissions proportionally based on temperature trends from 1979 to 2024.

**Figure 3:**
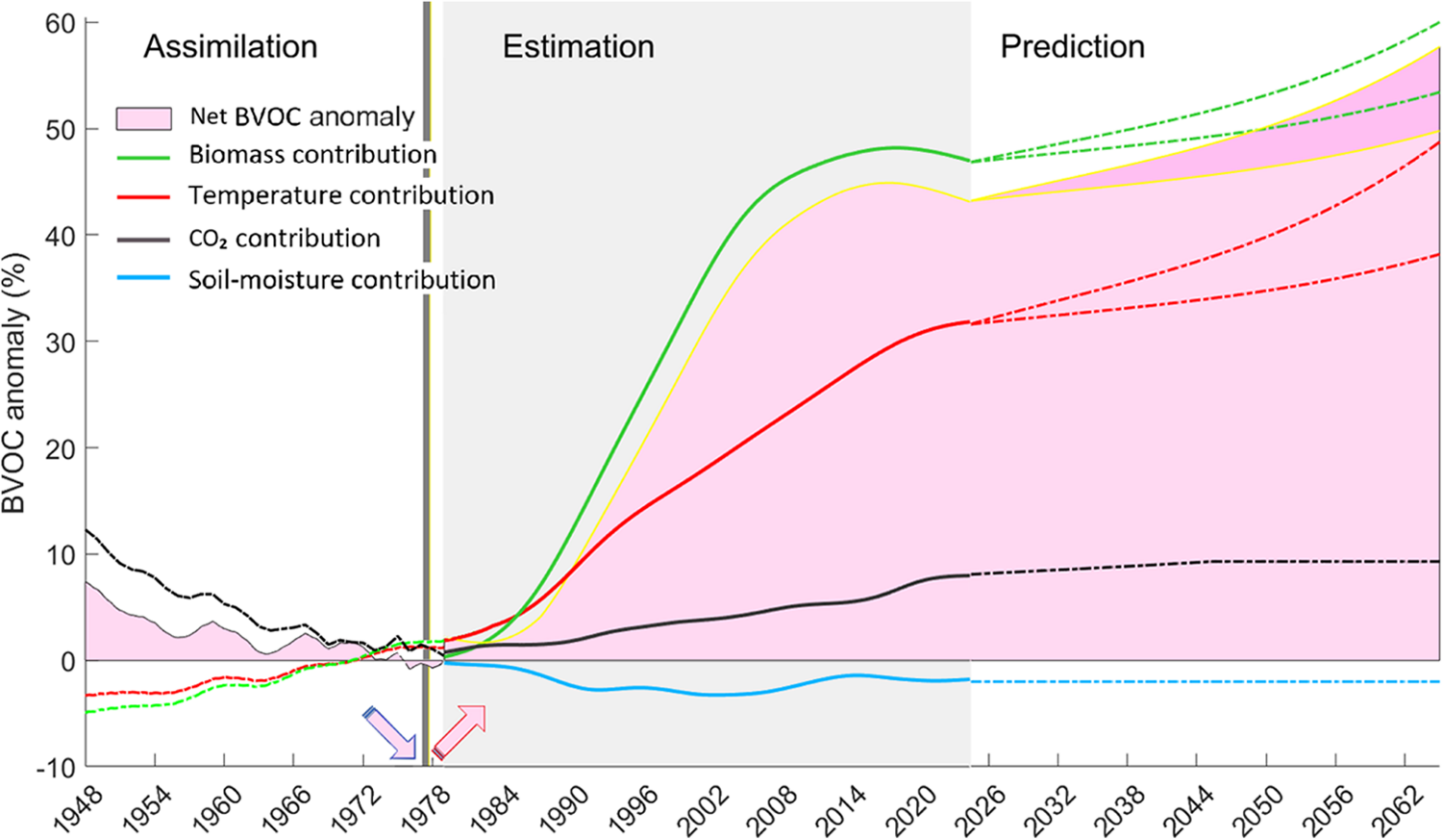
BVOC anomalies from 1948 to 2065, including historical data and projections. The gray-shaded area indicates the period when detailed remote sensing data were utilized, and the black line marks the year when the trend reversed.

Additionally, during 1945–1984, CO_2_ concentrations in Germany increased by approximately 180% (Figure S14), contributing to a decrease in BVOCs emissions. Assuming no significant anthropogenic interference—such as that which dominated BVOCs emissions increase between 1987 and 2005, temperature and CO_2_ concentration have been the principal factors controlling BVOC emissions in the 1970s and earlier. A stationary temperature coupled with stable CO_2_ levels in the 19th century (Figure S14) caused BVOC emissions to remain stable. From 1900 to the late 1970s, except for a few years post-World War II, CO_2_ levels increased rapidly, while temperature rose more slowly, leading to a decline in BVOC emissions. Since 1978, the sharp rise in global warming, coinciding with worldwide efforts to reduce CO_2_, has reversed this trend in Germany, resulting in increased BVOC emissions. Based on historical temperature and CO_2_ time series, the lowest BVOC emissions in Germany since the industrial revolution likely occurred around 1978 ± 1 year (Figure 3).

Looking ahead, we considered global warming scenarios from the Intergovernmental Panel on Climate Change (IPCC^65^) and Regional Climate Projections for Europe (RCP8.5^66^). Germany, identified as one of the regions with the most significant temperature increases globally (NOAA Repor^67^), may experience a rise of up to 5.5 °C compared to preindustrial levels by the end of the century. However, the influence of the CO_2_ concentration on BVOCs emissions is expected to diminish in the coming decades, particularly as Germany aims to achieve carbon neutrality by 2045 under the Climate Protection Act. Assuming minimal anthropogenic interference and negligible land cover changes, our projections suggest that, depending on future temperature and CO_2_ scenarios, emission rates will exhibit an increasing trend of 0.7% to 1.2%/year over the coming decades (Figure 3).

## 4 Evaluation and Assessment of Results

Currently, long-term measurements or assimilations relevant to BVOCs dynamics over decades are not available for Germany or other regions. However, remotely sensed measurements of Total Column Ozone (TCO) and Formaldehyde (HCHO) can serve as indicators of variations in BVOCs emissions, with each offering unique advantages and limitations. A summary of the evaluation process is presented in this section, and more details are appended to the Supporting Information (Text S4).

**Figure 4:**
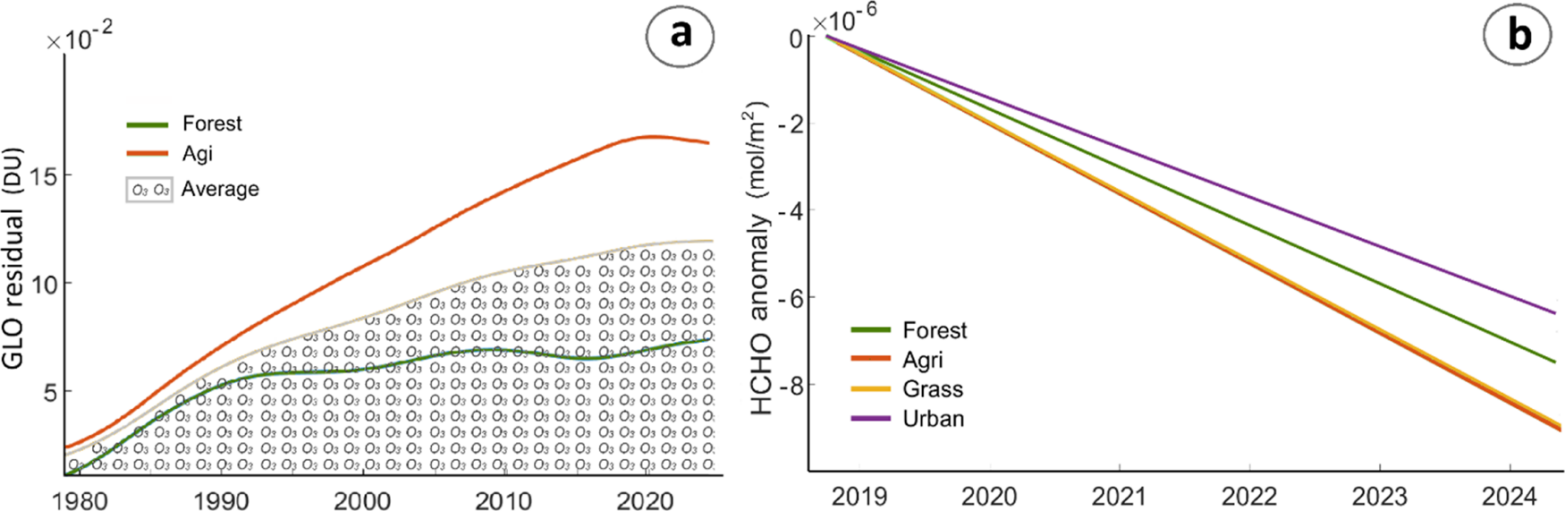
Time series of ground-level ozone over forest and agricultural areas (a) and trends of HCHO over three vegetation classes and urban areas (b).

Satellite-based total column ozone (TCO) time series have been available since 1978, provided by NASA/GSFC through the Total Ozone Mapping Spectrometer (TOMS)^68^ and the Ozone Monitoring Instrument (OMI). The lower portion of TCO, particularly ground-level ozone, is strongly influenced by the BVOCs. By analyzing TCO time series over adjacent seas, we subtracted the stratospheric component of TCO. After accounting for differences in emission rates, the average ground-level ozone from forested and agricultural lands demonstrated a strong correlation with modeled BVOCs anomalies (Figure 4a). HCHO is another important indicator of BVOC emissions. While only a small fraction of TCO may reflect BVOC influence, the entire HCHO vertical column is closely linked to BVOCs, making it an especially valuable variable for analysis. Vertical column HCHO has been directly measured by ESA’s Sentinel-5P satellite since October 2018. Our analysis revealed that grasslands and agricultural areas show larger decreases in HCHO compared to forests (Figure 4b), aligning with NDVI trends. In urban areas, HCHO levels are biased by additional VOCs induced by vehicles (Figure S19). Furthermore, urban areas exhibit smaller decreases in HCHO compared to other land cover types (see details in Text S4).

Another approach to evaluate the results is to compare them with findings from similar studies. Wang et al.^37^ modeled global variations in isoprene emissions from 2001 to 2020 using the MEGAN-3.2 model. They estimated a trend of +0.37% per year in Europe, attributing this increase equally to biomass changes and meteorological factors. To compare our results with theirs, we doubled their isoprene emissions and subtracted our CO_2_-induced contribution, which was not included in their study. Our adjusted estimate is 0.04% (∼10%) higher than theirs.

Lastly, to provide a level of confidence in our findings, we analyzed the overall accuracy of the contributors to BVOCs emissions. Among the four main contributors, biomass and temperature, which account for 76.5% of the total variation, are robust indicators. The studies agree on both the direction (positive/negative) and magnitude of the impacts that biomass and temperature have on BVOCs emissions. Importantly, these impacts can be measured independently for each contributor. Soil moisture has more variable relationships with BVOC emission depending on factors such as plant species and soil characteristics; However, they show a positive relationship in moderate soil moistures.^69^ The impact of CO_2_, however, remains the most challenging to assess. Specifically, long-term studies of the relationship of the CO_2_–BVOCs as an independent factor are lacking. Relying on the MEGAN model, and on the other studies in global scale,^37^ we estimated CO_2_’s contribution using the common approach; meanwhile, we assigned a wider range of error probability to CO_2_’s contribution due to these uncertainties. Accordingly, to reflect the sensitivity of the total anomaly to these contributions, we approximated an error margin of 3% for biomass, 3% for temperature, 10% for soil moisture, and 20% for CO_2_. Within these anticipated margins of error, the estimation of anomalies may vary by 7.34% (Figure S16) giving the final BVOCs anomaly equal to 53.66 ± 3.67%.

## 5 Discussion

When assessing the impacts of BVOCs emissions on the environment, comprehensive information on spatiotemporal dynamics is crucial. It will allow understanding the current status, predicting of future trends, and, consequently, development of strategies to mitigate the adverse effects of BVOCs on air quality and climate. BVOCs emissions rates and the types thereof are frequently discussed in literature.^70−76^

The study aimed at long-term national-scale BVOC dynamics (1790 to 2065), prioritizing large-scale trends over small-scale details. Due to significant data limitations and the inherent complexity of BVOC estimations, a direct approach—prone to uncertainties up to 40%—was avoided. Instead, an anomaly based methodology was adopted to reveal trends effectively while balancing data constraints and practical applicability over extensive spatiotemporal scales. The methodology intentionally excludes excessive detail, such as species-specific emissions or seasonal variations, as such precision would require unavailable high-resolution data sets and overly complex models. This generalization is reasonable, as small variations (e.g., trends of −0.11% and 0.05 °C in soil moisture and temperature, respectively) can be approximated without loss of accuracy. Thus, the approach is not an oversimplification but a deliberate strategy to capture BVOC long-term dynamics while overcoming data challenges. It ensures robust insights into national-scale trends despite unavoidable generalizations.

Remote sensing is still a relatively new approach for measuring the chemical composition of the atmosphere including BVOCs. Furthermore, if advanced sensors became able to directly measure BVOCs, they would still need to utilize proxies for simulating the past status of atmospheric components. Nevertheless, for complex parameters, including air chemical components, if the anomaly detection is not more important than quantification of the component concentration, they are equally important. We first fulfilled a detailed spatiotemporal investigation for the period in which spatial observations were available: 1979 to 2024. We also employed this methodology to simulate the variation in BVOCs emissions for several decades before and after 1979–2024. In order to determine proxy indicators for the state of BVOCs which were also suitable for remote sensing approaches, we reviewed and ranked several biological and abiotic environmental factors that may indicate changes in BVOCs emissions. These factors were then compiled into five maximally independent factors with distinct measurable impacts.

We benefited from the strengths of the two common VI-based and biomass-based approaches. To account for the different biomass capacities of each study site, floral assemblages were classified as forest, agriculture, or grassland. For agricultural land, crop diversity is also considered. We selected NDVI to estimate biomass, the efficacy of which was enhanced by our categorization of floral. Future studies may benefit from creating a hybrid NDVI-LAI to harness the inferential power of both indices simultaneously. For other researchers modeling biomass, it is important to note that the majority of models presented in the literature are fitted to relate the spatial distribution of biomass to VI, and thus are not applicable for modeling temporal dynamics.

Our result showed that from the start of the industrial revolution to the end of the 19th century BVOCs emissions were stable in Germany and then declined after 1900. In the late 1970s, this trend reversed and BVOCs emissions started to increase. We therefore chose 1978 as the reference date to compare emission rates. The results show that since 1978, the BVOCs emissions have increased by 53.66%. The highest increase was observed between 1987 and 2005. We found that the increase of BVOCs emissions from agricultural areas was dominant (58.4%). As agricultural land would not occur without human interventions in the landscape, we are confident in asserting that the largest increases in BVOCs emissions were caused by humans. In other words, if agricultural development had not occurred, then BVOCs emissions would only have increased by 27.14% in Germany. Although this is based upon the assumption that agricultural biomass (net crop production) is not influenced by the climate change. Nevertheless, any increase in agricultural productivity stemming from modern agricultural practices can still be considered an anthropogenic impact. We estimated an increase (11.26%) in seasonal fluctuations of the BVOCs emissions since 1978. Although readers should note that this seasonal increase is already included in our calculation of the 53.66% total increase. It is not an additional increase. We observed the largest increases in the eastern-central Germany, as well as in all urban areas. However, the scope of our study did not allow us to consider VOCs emissions from vehicles, which may also have increased. The presence of HCHO, for instance, is strongly associated with the scale and prevalence of hydrocarbon powered vehicles.^72^ Result confidence analysis showed that while biomass and temperature show robust and consistent impacts on BVOCs emissions, soil moisture, and CO_2_ contributions are more variable, particularly CO_2_ due to limited long-term studies. Our final estimation of BVOCs emissions, considering these factors, may vary by up to 7.34%, reflecting the anticipated margin of error.

The results were successfully evaluated using remotely sensed Total Column Ozone (TCO) and Formaldehyde (HCHO). The comparison provides an insight into the status of postpandemic HCHO in urban areas. Analyzing a time series of TCO over forest and agricultural lands led to the disclosure of the difference in the ground-level ozone production over these vegetation types. Agreement between the estimated ground-level ozone and the BVOCs interannual variations highlights the potential of TCO for BVOCs modeling. When compared to similar research on European BVOCs emissions, the similarity and closeness of the results is promising. Working from current global warming projections and with the assumption that there will be no further substantial anthropogenic interference, the rate of BVOCs emissions in Germany will continue to increase between 0.7% and 1.2% per year over the coming decades.

This study highlights the significance of anthropogenic influences, particularly agriculture, on the substantial increase of the BVOCs emissions. These insights should prompt policymakers to invest in advanced monitoring technologies for direct BVOCs measurement, enhancing the accuracy of future assessments and improving the understanding of their impact on air quality and climate. It also necessitates to incorporate BVOCs in climate action plans in Germany and develop targeted mitigation strategies such as reducing excessive biomass burning or optimizing crop selection to minimize BVOCs emissions. The methodology, which combines VI-based and biomass-based anomaly detection, can be adopted globally to create comparable data sets and improve the reliability of BVOCs emission modeling, fostering collaboration on climate change mitigation. By addressing these findings, the study informs both national and international efforts to tackle climate change and air quality challenges, emphasizing the urgent need for a multifaceted and evidence-based approach.

## Supporting Information Available

Presentation of the study area; PFT classification (Figure S1); analysis and ranking of influencing factors on BVOCs emissions; biomass modeling; evaluation and assessment of results; time series of: vegetation indices (Figures S2 and S3), precipitation, soil moisture and cloud cover (Figure S6), CO_2_ concentration (Figure S7); agricultural crops (Figure S8); time series of temperatures (Figures S10 and S13), precipitation (Figure S12), and long-term temperature and CO_2_ anomalies (Figure S14); seasonal fluctuations of vegetation indices (Figure S11), and error margins and accuracy of BVOCs emissions (Figure S20) (PDF)
