# Supplementary material for: Modeling Long-Term Dynamics of Biogenic Volatile Organic Compounds (BVOCs) in Germany Based on Major Precursors
Source: Environ Sci Technol. 2025 Feb 26;59(9):4587–96. doi: 10.1021/acs.est.4c14418 (PMC11912315; doi:10.1021/acs.est.4c14418)
Supplement: Supplementary file 1 — es4c14418_si_001.pdf [file es4c14418_si_001.pdf]

Supplementary Information for:

Modeling Long-Term Dynamics of Biogenic Volatile Organic Compounds (BVOCs) in Germany Based on Major Precursors

Ayoub Moradi[1*], *Temesgen Alemayehu Abera*[1], *Elliot Samuel Shayle*[1], *Mohammed Ahmed Muhammed*[1], *Dirk Zeuss*[1]

[1]Department of Environmental Informatics, Faculty of Geography, Philipps University of Marburg, Marburg, 35032, Germany

*corresponding author: Ayoub Moradi moradiay@geo.uni-marburg.de

Summary of supporting information:

Number of texts: 4

Number of tables: 1

Number of figures: 20

List of references

Content

Text S1. Study Area

Text S2. Individual Influencing Factors on BVOCs Emissions

Text S3. Biomass Modeling

Text S4. Evaluation and Assessment of Results

Table S1. Rate of unchanged pixels across different land cover classes in Germany

---

Figure S1. PFT classification generated from MODIS-derived vegetation indices using Random Forest algorithm.

Figure S2. Trends of NDVI and LAI across three vegetation classes in Germany.

Figure S3. Time series of monthly NDVI and LAI averages across Germany.

Figure S4. Time series of precipitation, soil moisture and cloud cover, averages across Germany.

Figure S5. Time series of CO_2 concentration, global average, Europe average and Germany average.

Figure S6. Trends of all factors affecting BVOCs emissions. Red and blue arrows indicate increases and decreases in BVOC emissions, respectively (normalized trends).

Figure S7. Trends of independent factors affecting BVOCs emissions (normalized trends).

Figure S8. The coverage area of each crop in past eight years (top), and the area percentage from the total, with error-bars indicating dispersion between years (bottom). Colors in the top panel are only for a better presentation.

Figure S9. Regression model between NDVI and biomass in forest and agricultural areas.

Figure S10. Time series and trends of maximum and minimum temperatures in Germany.

Figure S11. Seasonal fluctuation of NDVI, LAI and FAPAR in Germany (normalized values).

Figure S12. Time series of monthly total Precipitation, averages across agriculture and forests in Germany.

Figure S13. Time series of monthly mean temperature (top) and temperature trends across two epochs (bottom), averages over agriculture and forests in Germany.

Figure S14. Temperature and CO_2 anomalies in Germany since 1790. The grey line indicates the date before which historical data have been used.

Figure S15. Sample points over Germany and adjacent waters for extraction of time series of Total Column Ozone.

Figure S16. Time series of Total Column Ozon in Germany and adjacent seas (Dobson Units).

Figure S17. A: time series of TOC over forest and agriculture, B: stratospheric ozone removed, and, C: accounted for the forest's larger emission capacity.

Figure S18. A: sample points over Germany, B: time series of HCHO over four land covers: forest, agriculture, grassland and urban areas.

Figure S19. Trends of HCHO: actual quantities (a), and adjusted to zero (b).

Figure S20. Margin of error for each BVOCs contribution and the resulting accuracy of BVOC anomaly.

References

STUDY AREA

Germany, located in Central Europe, spans approximately 357,000 km² and encompasses a variety of ecosystems, ranging from low-lying northern coastlines to the forested uplands of the Central Uplands, and the Alpine regions in the south. Germany is characterized by a diverse range of ecosystems, shaped by its varied topography, climatic conditions, and land use. Germany's climate, predominantly temperate, supports a mix of natural vegetation types, ranging from deciduous and coniferous forests to grasslands and wetlands. Forests, accounts for about 33% of the country’s total area. Beech (Fagus sylvatica) forests are characteristic of Germany's natural vegetation, representing one of the most widespread and ecologically significant forest types in Europe. Other notable tree species include oak (Quercus spp.), spruce (Picea abies), and pine (Pinus sylvestris), which are often found in mixed or pure stands. Germany is also one of Europe's leading agricultural producers, with around 50% of its total land area dedicated to agricultural use. The country’s farming landscape is diverse, ranging from large-scale arable farms in the north to smaller, mixed farms in the south. Key crops include cereals such as wheat, barley, and rye. Vegetation cover in Germany, where

urbanization and industrial activities contribute to air pollution, plays a significant role in influencing air quality through its ability to absorb pollutants, release BVOCs, and regulate microclimatic conditions. Balance of these effects varies with vegetation type, land-use patterns, and climatic factors. Germany's vegetation is strongly influenced by centuries of human activity, including agriculture, urbanization, and forestry. The country's extensive vegetation coverage, thus, makes it an ideal location for studying BVOCs.

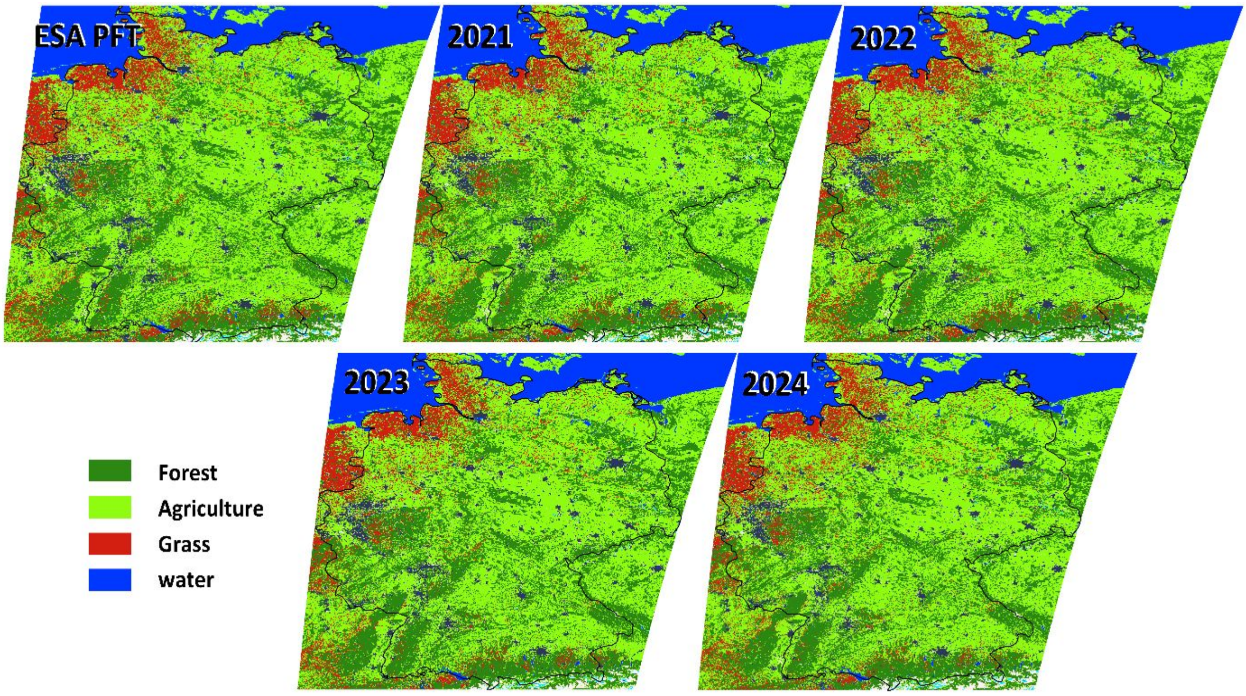

Figure S1. PFT classification generated from MODIS-derived vegetation indices using Random Forest algorithm.

Table S1. Rate of unchanged pixels across different land cover classes in Germany

Land cover	Grass Man	Tree NE	Tree BD	Grass Nat	Bare land
Area (% of Germany)	51	17.6	14.4	9	1.2
Unchanged pixels (% of the whole)	95.4	95.5	97.2	93	99.9

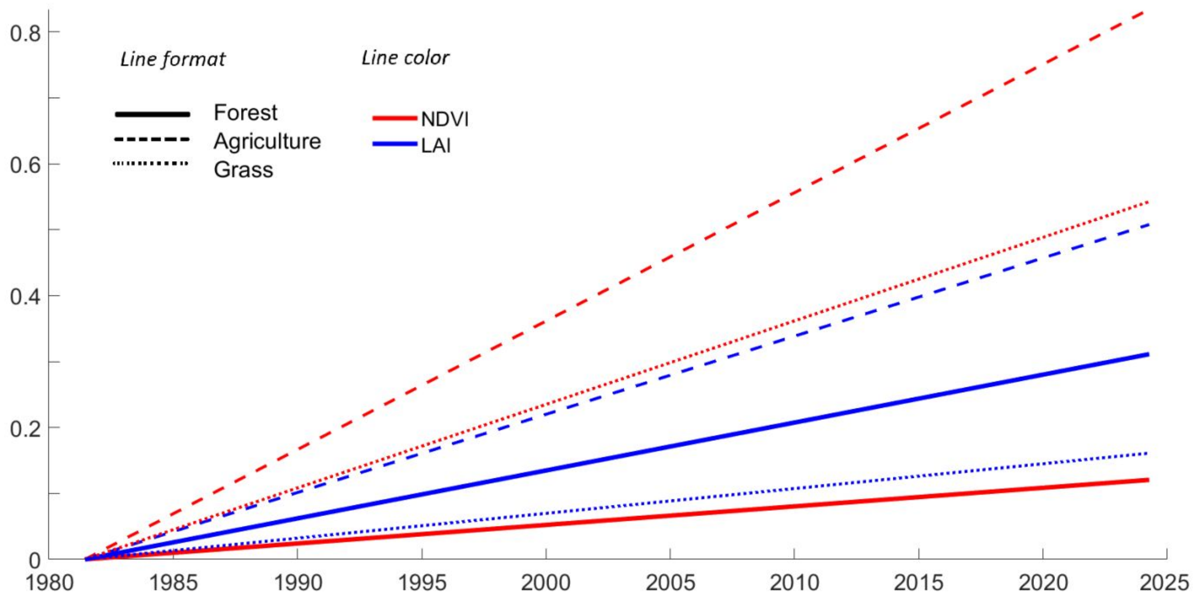

Figure S2. Trends of NDVI and LAI across three vegetation classes in Germany.

INDIVIDUAL INFLUENCING FACTORS ON BVOCS EMISSIONS

Biological Factors

1- Vegetation cover

Terrestrial vegetation is the primary source of BVOCs emissions[7], and thus, plays a dominant role in biological factors. BVOCs emissions are closely linked to plant photosynthetic rates, with higher photosynthetic activity leading to increased precursor availability for BVOC synthesis. As discussed earlier, total emissions are calculated by multiplying the emitter (i.e., vegetation cover) by the emission factor. Vegetation covers, as the emitters, are usually derived from land cover classifications, specifically from plant functional type (PFT) classes. In addition to static vegetation classes, which represent plant species and their spatial extents, dynamic characteristics of vegetation cover are essential for updating the time-variable scaling factor. Vegetation cover is typically studied in remote sensing through spectral criteria known

as vegetation indices. In this study, we utilized the Normalized Difference Vegetation Index (NDVI), Leaf Area Index (LAI), and the Fraction of Absorbed Photosynthetically Active Radiation (FAPAR). NDVI provides insights into greenness, which can be roughly translated to vegetation extent within a pixel. LAI represents the total one-sided leaf area of plants per unit ground area, offering information about the amount of material in plant canopies, which is directly related to photosynthetic capacity. Accounting for illumination conditions, FAPAR represents the fraction of incoming solar radiation used for photosynthesis. Together, these indices provide valuable information about the density, health, and photosynthetic capacity of plants.

The monthly time series showed that all three indices have increased in Germany, with the lowest and highest increases observed in NDVI and LAI, respectively. An increase in vegetation indices may indicate a corresponding increase in BVOCs emissions. However, in the case of NDVI and LAI, interannual signals are stronger than the trends (Figure S3). This suggests that trends alone cannot capture all variations, and interannual fluctuations should also be considered.

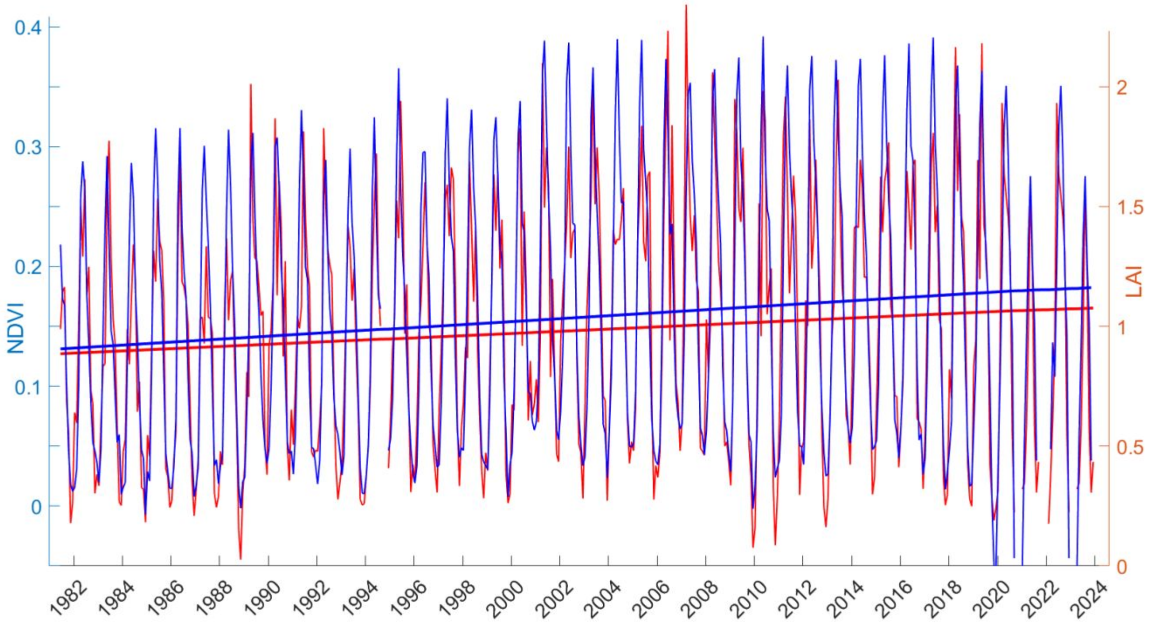

Figure S3. Time series of monthly NDVI and LAI averages across Germany.

2- Other biological factors

Livestock and soil microorganisms can partially influence the BVOCs emissions[8]. Studies have consistently highlighted that the contribution of animals to BVOCs emissions (less than 1%) is negligible compared to that of plants. Particularly, the Livestock contribution in BVOCs variations can be confidently disregarded. Soil microorganisms, on the other hand, contribute to BVOCs emissions, particularly in response to soil temperature and moisture conditions[9,10]. These microorganisms either produce BVOCs directly or modify plant emissions through biochemical interactions. The exact contribution of soil microorganisms to BVOCs emissions is highly case-dependent, as the microbial activity is influenced by various factors, including soil moisture, temperature, and the presence of organic matter. For example, during periods of organic matter decomposition, microbial activity and, consequently, BVOC emissions may increases[10]. Variations in soil BVOCs emissions are closely tied to plant phenology and

environmental factors, particularly soil moisture. These variations are largely reflected in the analyzed environmental factors, especially biomass, temperature, and soil moisture.

Environmental Factors

Solar energy is the primary source of energy for Earth. By regulating light and heat, the sun drives periodic variations in BVOC emissions, evident in diurnal and seasonal cycles. However, the sun's influence on BVOC emissions may also involve long-term variations due to secondary factors. This section analyzes variations in solar radiation flux and the resulting environmental effects.

1- Downward Short-Wave Radiation (SWR)

Downward short-wave Radiation flux at the surface, often referred to as surface solar radiation, is the amount of solar energy received per unit area at the Earth's surface measured in watts per square meter (watts /m²). The solar angle—varying with latitude, time of day, and season—primarily affects the intensity and duration of solar radiation at the Earth's surface. However, as SWR passes through the Earth's atmosphere, it can be absorbed, reflected, or scattered by gases (e.g., Ozone), water vapor, clouds, and aerosols. Time series analysis showed that the average SWR in Germany ranges from 35 to 220 (kWh/m²/month). Since 1979, SWR has shown a positive trend, with an increase of 28 kWh/m²/year, which corresponds to approximately 15% of its annual fluctuation. This increase is recorded the highest rate of increase among the studied factors. SWR impacts the BVOCs emissions directly through the

photosynthesis process, and indirectly by affecting temperature, precipitation, and plant growth. Therefore, an increase in SWR results in an increase in BVOCs emissions.

2- **Temperature (T)**

BVOCs emissions are strongly influenced by temperature. Any rise in temperature increases the emission rates of most BVOCs due to enhanced enzymatic activities and increased volatility of these compounds[11,12]. Common BVOCs models, e.g., the MEGAN, include specific adjustments in their algorithms to account for temperature variations. In line with this, the mean temperature in Germany has shown a positive trend, with an increase of 1.9˚C at 2 meters above ground level, likely due to increased solar radiation.

3- **Precipitation (P)**

Adequate rainfall can enhance plant metabolic activity, leading to higher production and release of BVOCs[13]. To a certain level, it can also increase soil BVOCs emissions. Conversely, by reducing temperature and light while increasing humidity, rainfall can also decrease BVOCs emissions. In addition, heavy rain and subsequent flooding can cause stress and physical damage to plants, potentially increasing their BVOCs emissions. Therefore, depending on conditions, rainfall can influence the BVOCs emission in both directions. Time series showed that the average rainfall ranges from ~10mm in winter months to ~80mm in summer months. The long-term trend shows a very slight decrease in rainfall (Figure S4a), which likely has a negligible effect on BVOCs emissions.

4- **Soil moisture (SM)**

Soil moisture has a multifaceted influence on plant BVOCs emissions, through its effects on plant physiology, temperature regulation and soil microbial activity. Adequate soil moisture generally supports regular BVOCs emissions. The depth at which BVOCs can be affected by soil moisture varies depending on several factors, including soil composition, microbial activity and root distribution. Most impacts occur near the soil surface (0-10 cm depth) where microbial activity and root density are highest. BVOCs can also be emitted from the rhizosphere (20-30 cm deep), which is the soil zone influenced by root secretions. Emissions from soil beyond 30 cm are generally less significant due to reduced microbial activity and lower organic matter content[14,15]. Data from the Volumetric Soil Moisture Content (VSMC) provided by the National Centers for Environmental Prediction (NCEP) Climate Forecast System (CFS) are estimated for 4 levels of depth from the surface: 5cm, 25cm, 70cm and 150cm. We analyzed the 25cm below the surface to account for maximal activity of soil organisms. Soil moisture in the 25 cm layer showed a decrease of about 3.5% since 1979 (Figure S4b).

5- Cloud cover (CC)

Cloud cover reduces both amount and spectral quality of solar radiation reaching the Earth's surface. Since BVOCs emissions are closely linked to photosynthetic activity, a decrease in light intensity can lead to reduced emissions rates. Additionally, clouds can have a cooling effect by reflecting sunlight, which further decreases BVOCs emissions. Time series showed that the average cloud cover has decreased from 48.6% to 43.8% (Figure S4c).

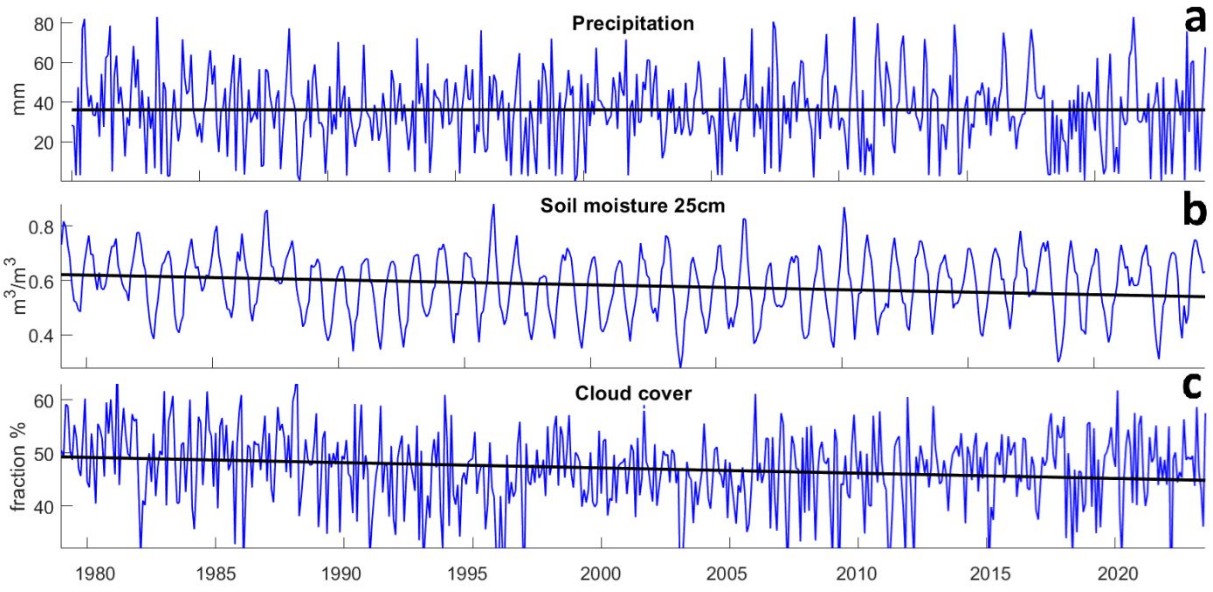

Figure S4. Time series of precipitation, soil moisture and cloud cover, averages across Germany.

6- CO_2 (Carbon Dioxide)

The effect of CO_2 concentration on BVOCs emissions involves a complex interaction. Lower CO_2 levels can limit the carbon available for isoprene production, leading to reduce the total emissions. According to certain studies, there is a positive short-term relationship between CO_2 levels and isoprene emissions of plants[16,17,18]. Meanwhile, a commonly cited interaction indicates on a negative relationship between BVOCs and CO_2 level in log-term[16,19,20,21,22,23,24,25].

The CO_2 time series obtained from the 'Our world in data' database[26] showed a significant trend in CO_2 emission in Germany. Since 1990s, factors such as reunification, the industrial modernization and the implementation of renewable energy incentives are leading to more efficient energy use and reduced dependence on coal (www.bundesregierung.de). Germany's ambitious climate policies, particularly through the Renewable Energy Sources Act (EEG), have resulted in substantial CO_2 emission reductions over the past decades. Contrary to the

global average increase, the time series indicates a significant decrease in CO_2 emission in Germany (Figure S5).

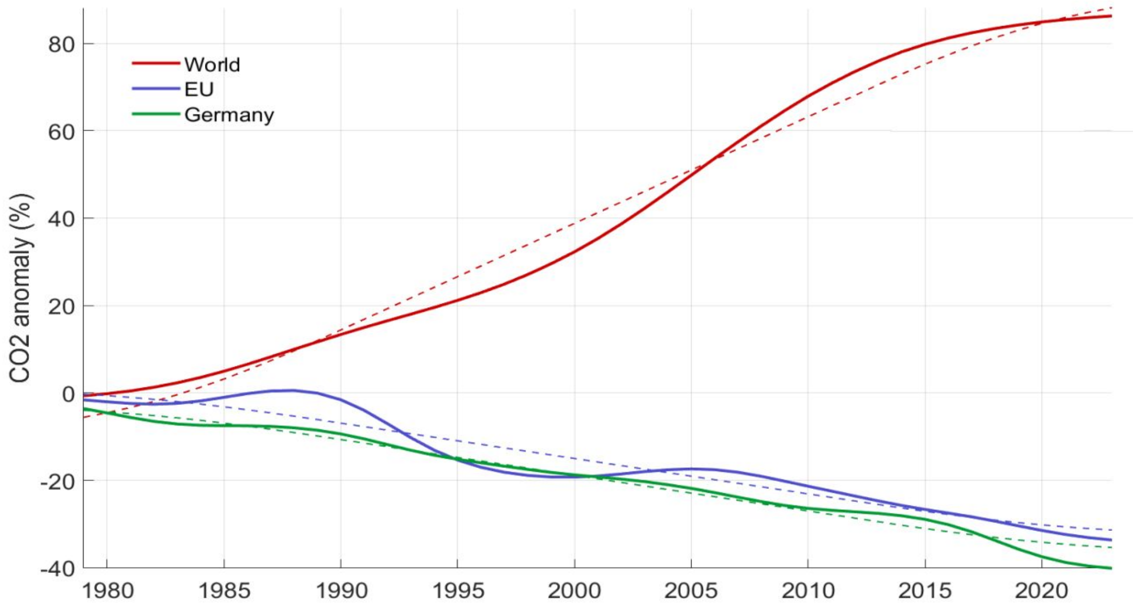

Figure S5. Time series of CO_2 concentration, global average, Europe average and Germany average.

7- Wildfire area (WF-A) and frequency (WF-F)

Wildfires can lead to significant emissions of BVOCs due to the combustion of vegetation[14]. The contribution of wildfires to BVOCs emissions can vary significantly depending on the region, the type of vegetation burned, and, the intensity and duration of the wildfire. Warming temperatures and drying climate are projected to increase the frequency and intensity of wildfires and lengthen fire seasons globally[27,28]. In areas with frequent and intense wildfire activity, such as certain forested regions in North America, Australia, and the Mediterranean, wildfires can be a major source of BVOCs. Although Germany is largely covered by vegetation, it is not among the regions with frequent wildfires. According to the data from the German

Federal Ministry for the Environment, Nature Conservation (www.bfn.de), as released by the Federal Ministry of Food and Agriculture (www.bmel-statistik.de), the most significant wildfires have occurred orderly on 1992, 2021, 2019 and 2018 (Figure S6). The 1992's wildfire in the forested Lüneburg Heath (northeastern Germany), lasted for several days. The data also showed an increase of wildfire frequency after 2018, comparing to the average over the last 35 years.

8- Other environmental factors

As a final environmental factor, volcanic activity can indirectly influence BVOCs emissions. Volcanic eruptions release a variety of gases and particles, e.g., sulfur dioxide (SO_2), carbon dioxide (CO_2) and hydrogen sulfide (H_2S). These emissions can affect plant physiology and depositing nutrients to the soil[29]. In addition, volcanic activity can alter temperature, light conditions, thereby affecting the BVOCs emissions. Monitoring of volcanic activity of active fields in Germany (in Eifel region) and in regional scale (e.g., in Italy, Spain and Island) helps assess its impact on BVOCs emissions. Normalized trends of the analyzed influencing factors are shown in Figure S7.

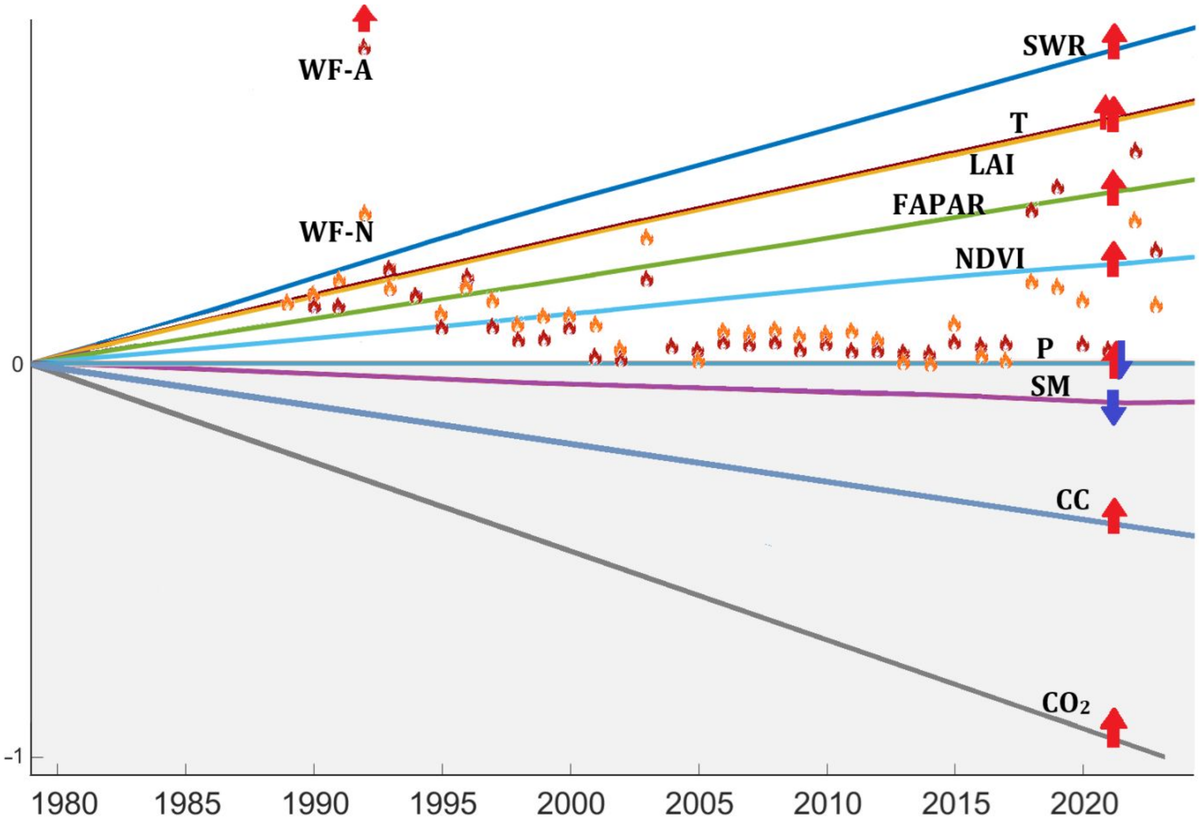

Figure S6. Trends of all factors affecting BVOCs emissions. Red and blue arrows indicate increases and decreases in BVOC emissions, respectively (normalized trends).

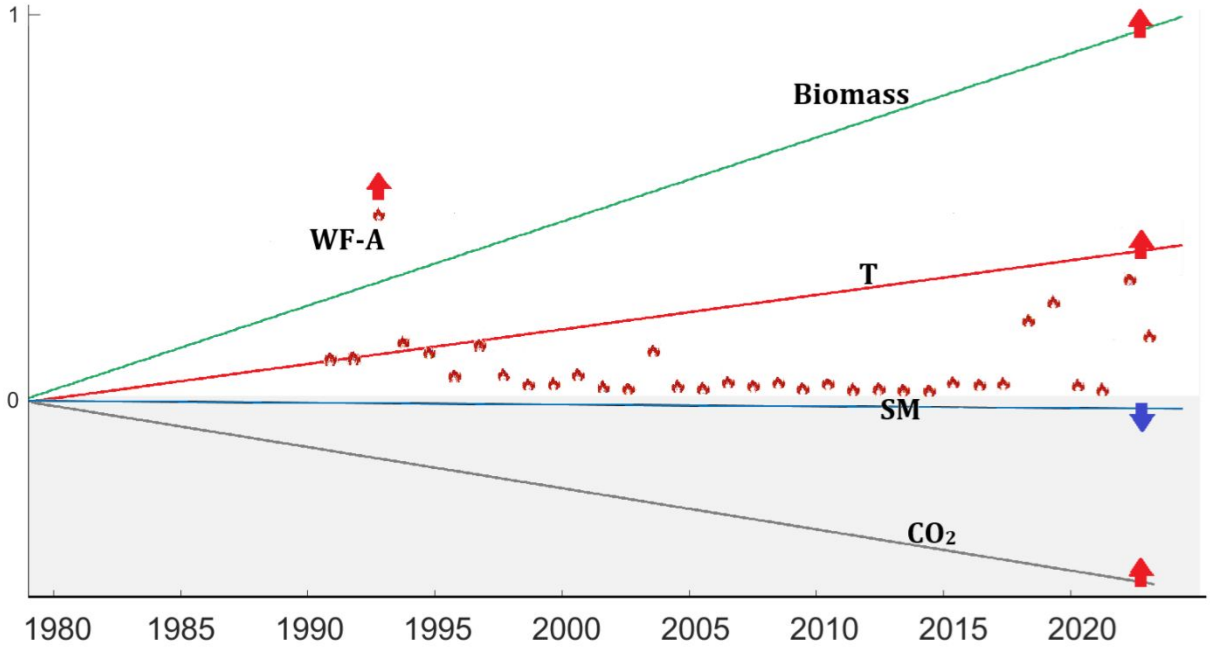

Figure S7. Trends of independent factors affecting BVOCs emissions (normalized trends).

BIOMASS MODELING

An analysis across three main vegetation classes reveals significant differences in the sign and slope of VI trends (Figure S2). The agricultural class showed the greatest increases in both NDVI and LAI, largely due to advances in agricultural practices over recent decades. Unlike the agricultural and grassland classes, the forest class exhibits a greater increase in LAI compared to NDVI. This is attributed to the larger biomass of forests, which allows for greater potential increases. However, the extent of forested areas has not expanded as much as the vegetation extent in the other two classes, resulting in a smaller slope of increase for NDVI. Accordingly, NDVI was prioritized to calculate biomass; because, as it provides better resolution for detecting biomass variations (see Figure S2). Although grasslands are relatively uniform, forests and agricultural areas, which together account for about 83% of the country's area, comprise diverse tree species and crops with varying biomass coefficients. We accounted for these subclasses to develop more accurate models.

1. Biomass Model for Agricultural Areas and Grassland

The variety and coverage of agricultural crops in Germany were analyzed using data spanning eight years. According to the statistics of the German Federal Statistical Office[1], wheat, corn, barley and rye were the most common crops from 2016 to 2023 occupying the top five ranks. Together, these crops accounted for approximately 77% of the cultivated land (Figure S8). The biomass coefficient for the agricultural category was calculated as a weighted sum of the

individual crop coefficients: wheat = 7.5, maize = 12.5, barley = 6, oilseed = 3, and other = 3.5 tons/hectare; leading to a weighted sum of 6.76 tons/hectare. An eight-year regression analysis (Figure S9) resulted in the following linear model:

Biomass Agriculture = 110 * NDVI + 5.6 (ton/hec)

Due to the general similarities between the biomass values of natural grasslands and agricultural lands, and the lack of specific biomass data for grasslands in Germany, this model was also applied to estimate grassland biomass.

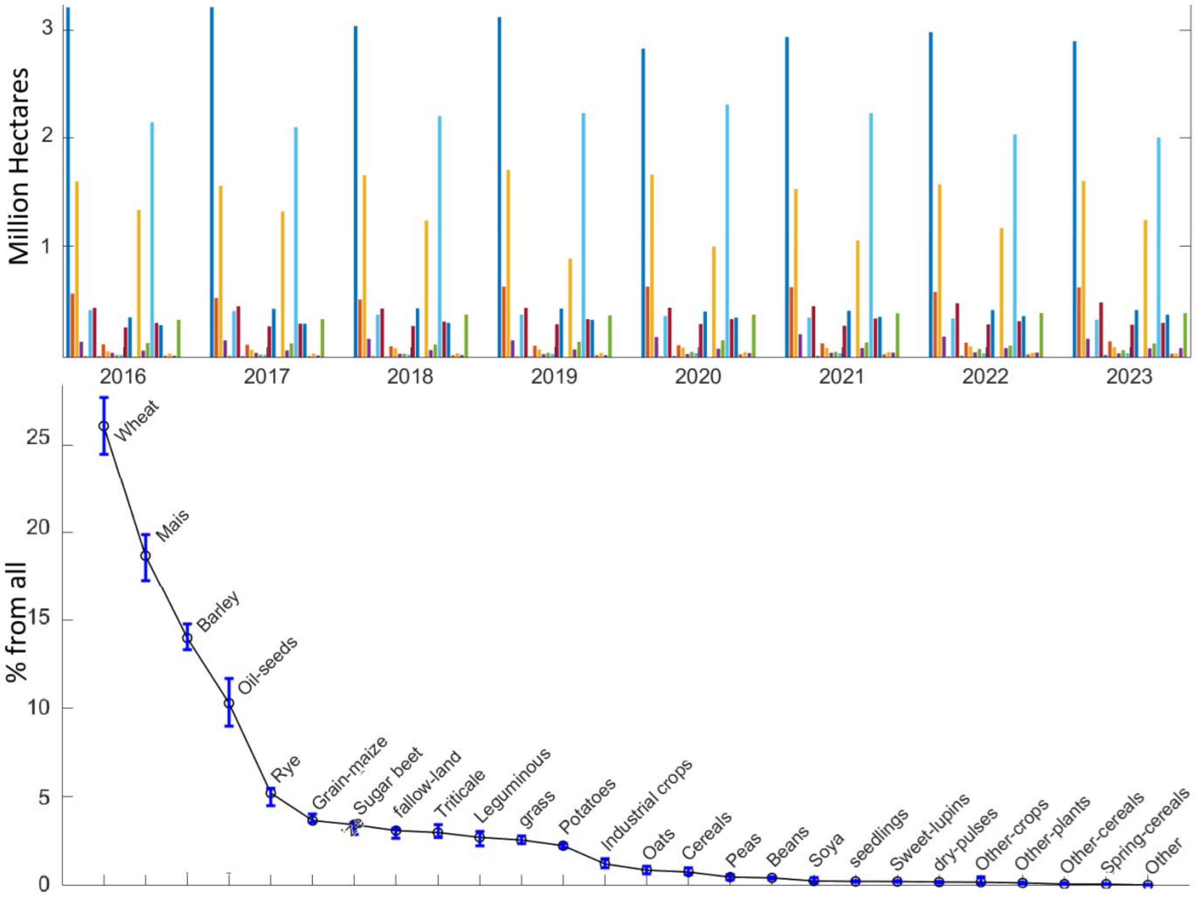

Figure S8. The coverage area of each crop in past eight years (top), and the area percentage from the total, with error-bars indicating dispersion between years (bottom). Colors in the top panel are only for a better presentation.

2. Biomass Model for Forest

In the absence of specific biomass records necessary to develop a direct model, forest biomass was estimated from Growing Stock Volumes (GSV) data of German forests published by FAO[2]. The GSV refers to the total volume of all living trees, typically excluding leaves and roots. The time series of forest biomass was calculated from GSV, wood density, and Biomass Expansion Factors (BEF) using a linear equation:

Biomass (ton/hec) = GSV (m^3/hec) * Wood Density (g/cm^3) * Biomass Expansion Factor

BEFs are identified to account for the biomass of the entire tree, including the parts that are not typically measured in volume. GSVs and corresponding wood densities are species-specific. The main tree species in German forests include Norway Spruce (27%), Scots Pine (24%), Beech (17%), and Oak (12%). According to data from the Federal Forest Inventory[3] covering 11 years (between 1990 and 2020), the wood densities for these species are 0.48, 0.55, 0.73, and 0.75 g/cm^3, respectively[4,5]. The BEF is determined as 1.4 for Norway Spruce, and 1.3 for Scots Pine, Beech, and Oak[6]. An 11-year regression analysis between biomass and respective NDVI values (Figure S9) yields the following model:

Biomass Forest= 1129 * NDVI + 21.86 (ton/hec)

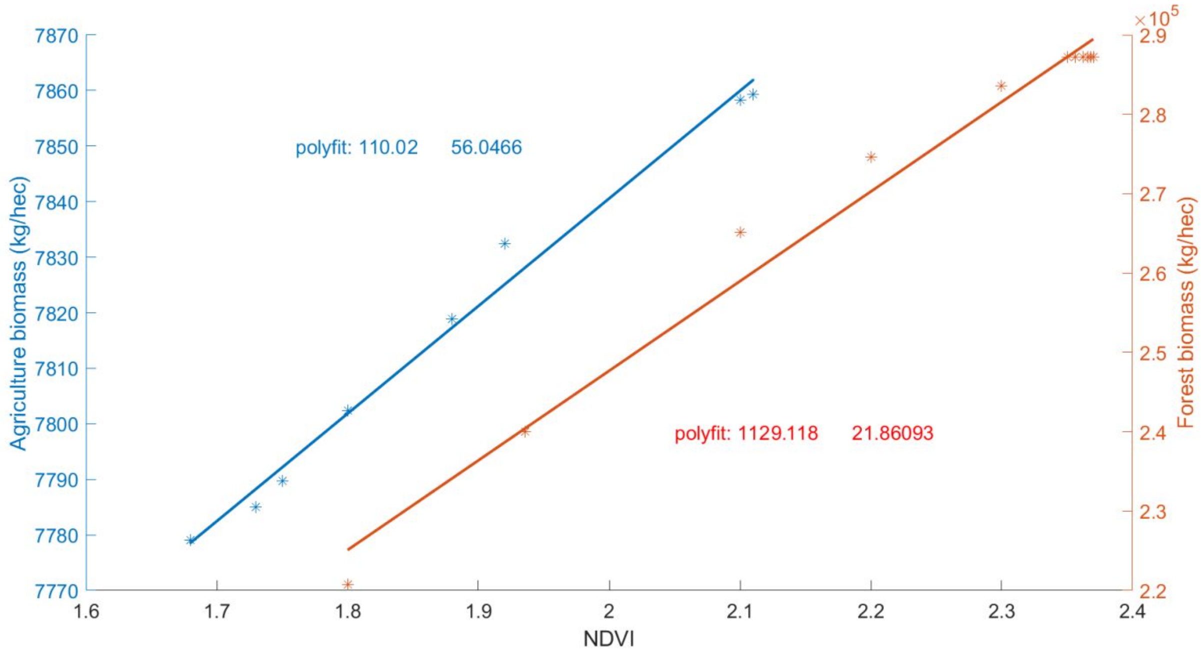

Figure S9. Regression model between NDVI and biomass in forest and agricultural areas.

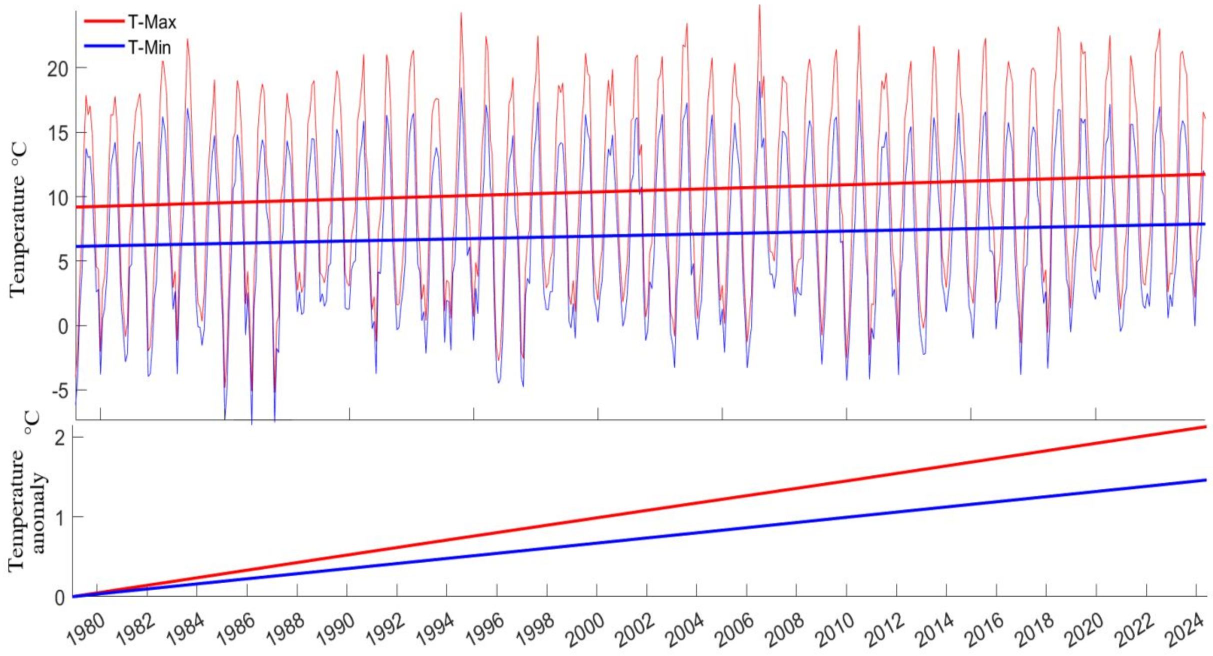

Figure S10. Time series and trends of maximum and minimum temperatures in Germany.

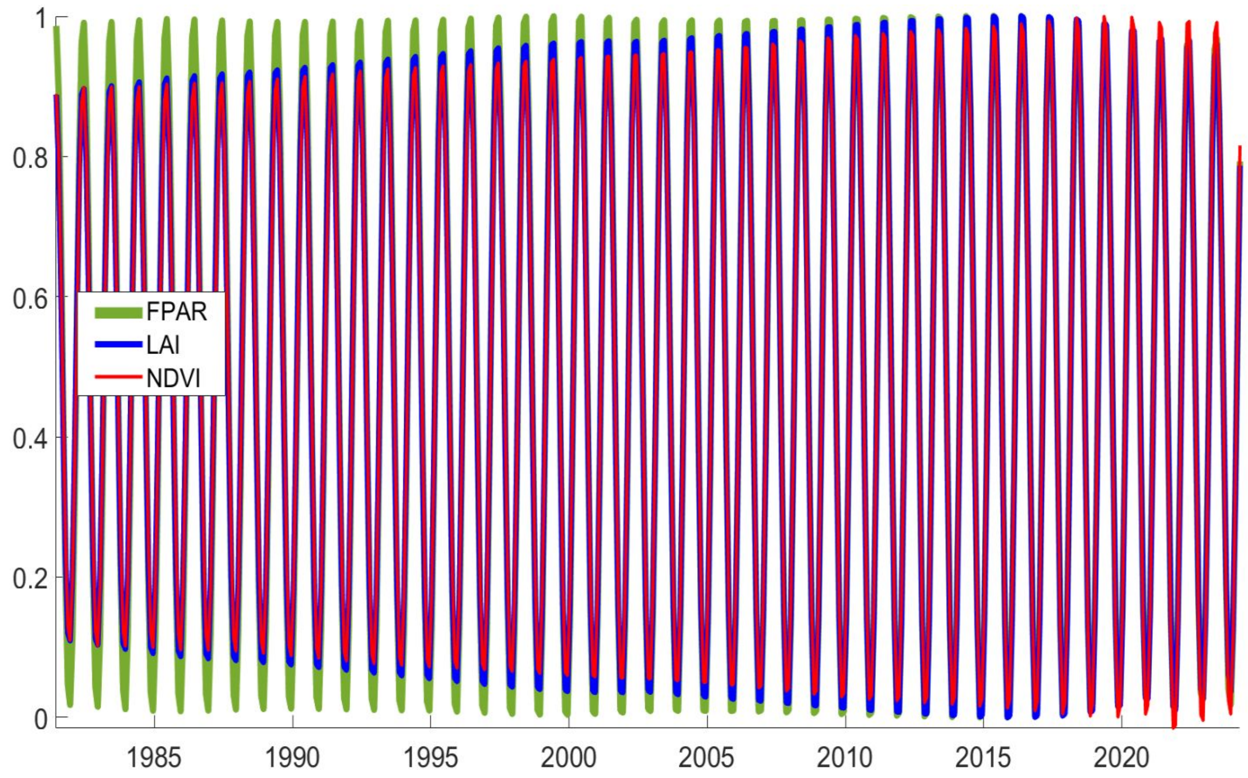

Figure S11. Seasonal fluctuation of NDVI, LAI and FAPAR in Germany (normalized values).

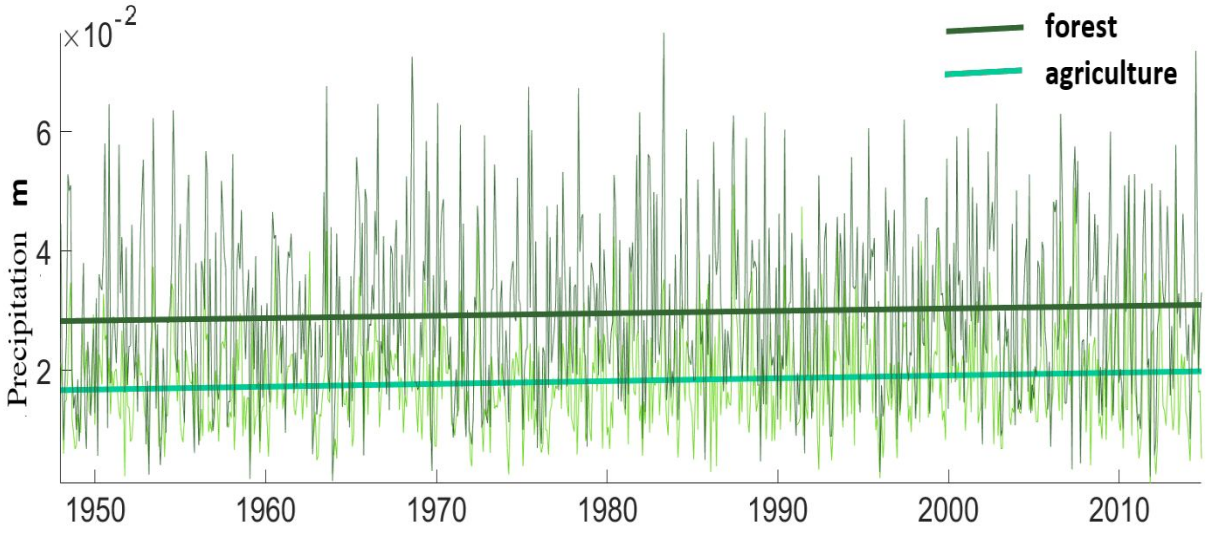

Figure S12. Time series of monthly total Precipitation, averages across agriculture and forests in Germany.

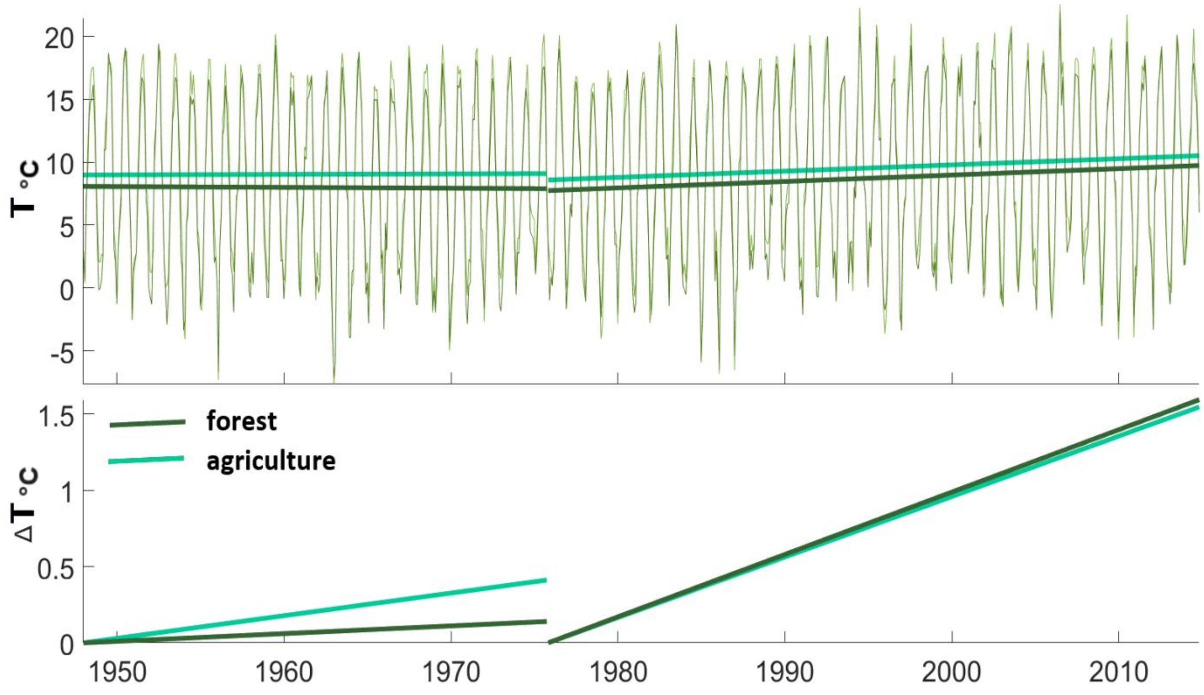

Figure S13. Time series of monthly mean temperature (top) and temperature trends across two epochs (bottom), averages over agriculture and forests in Germany.

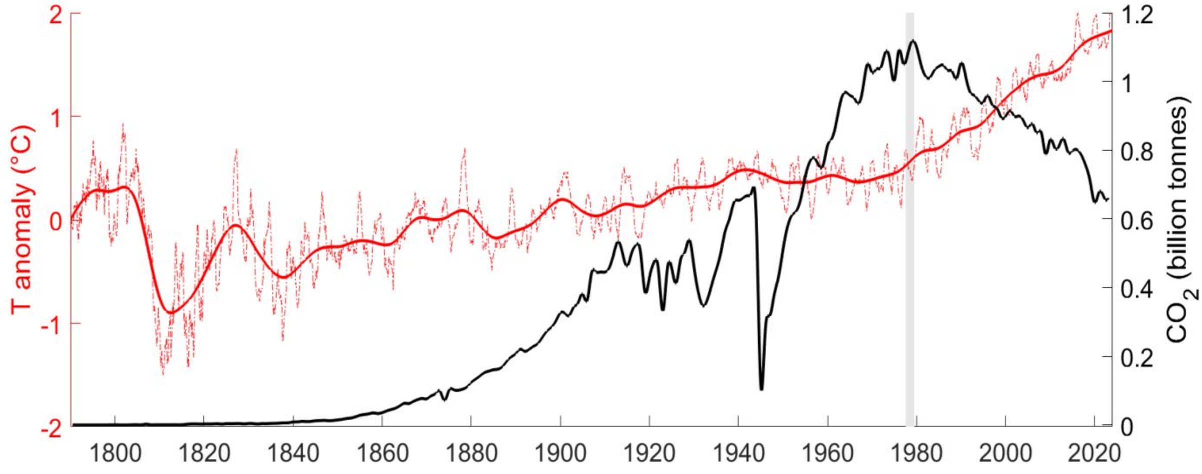

Figure S14. Temperature and CO_2 anomalies in Germany since 1790. The grey line indicates the date before which historical data have been used.

EVALUATION AND ASSESSMENT OF RESULTS

Long-term data relevant to BVOCs dynamics are not available. However, certain remote sensing atmospheric products can serve as indicators of variations in BVOCs emission. In this section we evaluate the results through remotely sensed measurements of Total Column Ozone (TCO) and Formaldehyde (HCHO), with each offering unique advantages and limitations. Another approach to evaluate the results is by comparing them to those provided by similar studies; This comparison is presented in the manuscript.

1. Evaluation Through the Total Ozone Column (TOC)

In the presence of nitrogen oxides (NO_2) and sunlight, BVOCs can contribute to the formation of Ground-Level Ozone (GLO) through a series of chemical reactions. Therefore, except for urban areas, where GLO may be influenced also by non-biogenic VOCs, there is a direct relationship between BVOC and GLO. We obtained Total Column Ozon (TCO) data from the NASA Goddard Earth Sciences Data and Information Services Center (GES DISC). These data are available in 1° by1° since 1978. Time series of TCO were collected for 180 regular grids across Germany and 150 regular grids over the surrounding waters (Figure S15). Averaged time series of TOC over Germany and adjacent seas are shown in Figure S16.

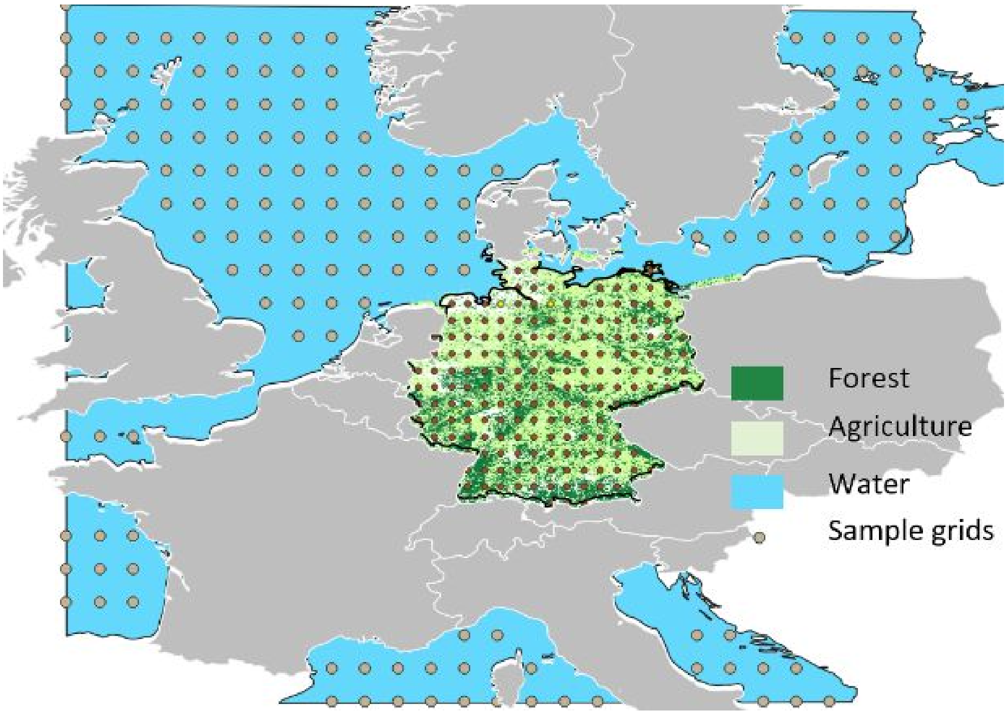

Figure S15. Sample points over Germany and adjacent waters for extraction of time series of Total Column Ozone.

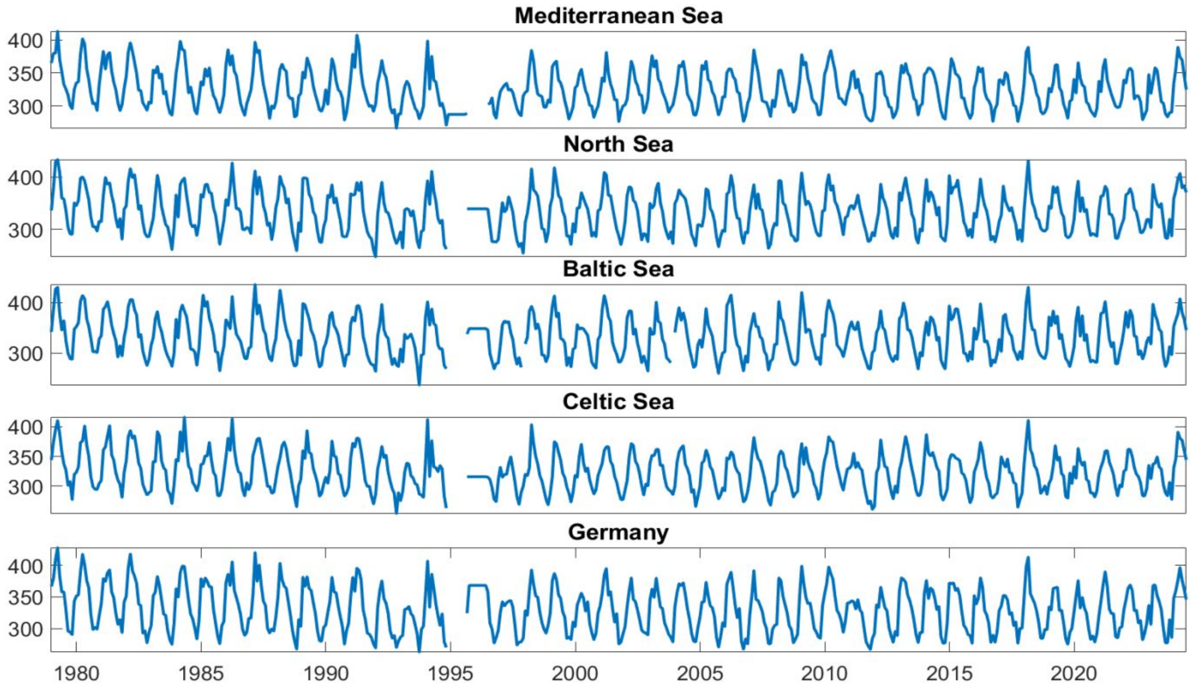

Figure S16. Time series of Total Column Ozon in Germany and adjacent seas (Dobson Units).

Among the land sample points, only those located within pixels consisting the purest forest and agriculture (>70%) were selected for further analysis. Supposing that TCO over waters are maximally free of biomass and BVOC impacts, they employed to calibrate land TCO's samples

in order to subtract the stratospheric ozone from TCO. This led to invert the trends from decreasing TCO (Figure S17, a) into increasing GLO residuals (Figure S17, b). The observed difference in trends between forested and agricultural areas indicates a larger increase in GLO for forests. This is attributed to forests' higher contribution to GLO in TCO compared to the smaller contribution from agricultural areas. To account for the differences in baseline BVOC emissions and to make the anomalies comparable, the time series data were normalized. Specifically, because forests produce significantly more BVOCs, the contribution of forest GLO to TCO is approximately three times larger than that of agricultural GLO. As a result, the forest GLO values were divided by three for normalization purposes (Figure S17, c). As the Figure clearly show the agricultural area has experienced a significantly larger increase in GLO which is influenced by the larger increase in biomass, consisting with the NDVI trends in Figure S2. The average of forest and agriculture shows a clear similarity with the BVOC anomaly (Fig 4, a and Fig.1 in the manuscript).

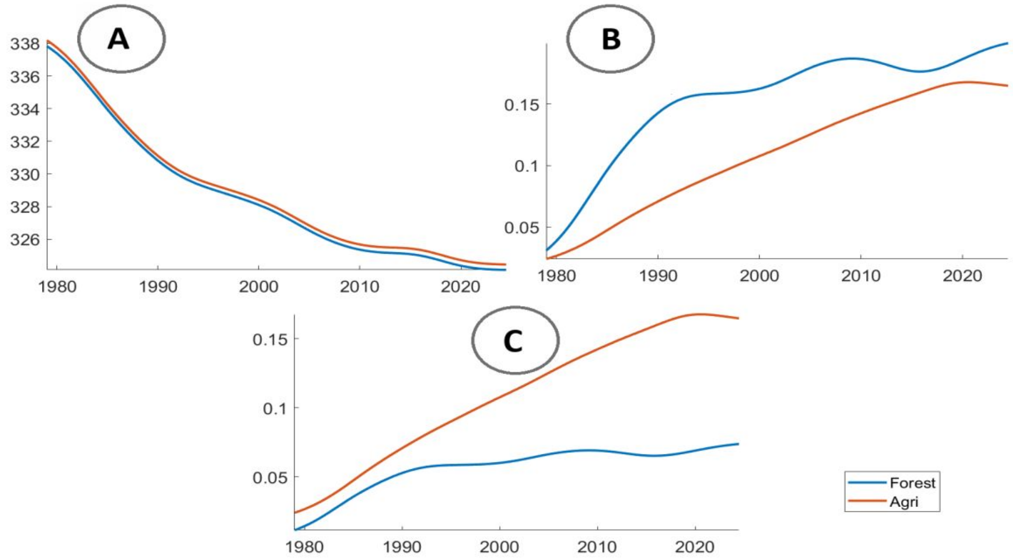

Figure S17. A: time series of TOC over forest and agriculture, B: stratospheric ozone removed, and, C: accounted for the forest’s larger emission capacity.

2. Evaluation Through Formaldehyde (HCHO)

Formaldehyde (HCHO) is a key atmospheric trace gas that plays a significant role in the chemistry of the troposphere. It is a secondary product formed during the oxidation of biogenic and non-biogenic VOCs. HCHO levels tend to peak during warm and sunny periods of day and year, when the BVOC emissions also peak due to the maximum photosynthesis and atmospheric photochemical reactions. While only a small fraction of total column ozone may indirectly reflect the influence of BVOCs, the entire vertical column of HCHO may directly be linked to BVOC emissions. Furthermore, the spatial resolution of HCHO measurements (1 km) far surpasses that of TCO (1 degree). These characteristics make HCHO particularly valuable parameter for studying biogenic contributions to atmospheric chemistry. Unlike TCO, which requires additional analysis to isolate its BVOC-related component, HCHO provides a more direct and comprehensive indicator of BVOC activity throughout the atmospheric column. This specificity enhances its utility in evaluating BVOCs emission patterns and their impact on air quality and climate. Since October 2018, vertical column HCHO has been directly observed with high spatial and temporal resolution by ESA's Sentinel-5P satellite, offering a powerful tool for monitoring BVOC dynamics and their interactions with other atmospheric constituents on both regional and global scales. HCHO time series over 180 regularly distributed grids have been collected for 2018-2024. Samples were grouped in 4 classes based on land covers, including forest, agriculture, grass and urban areas (Figure S18).

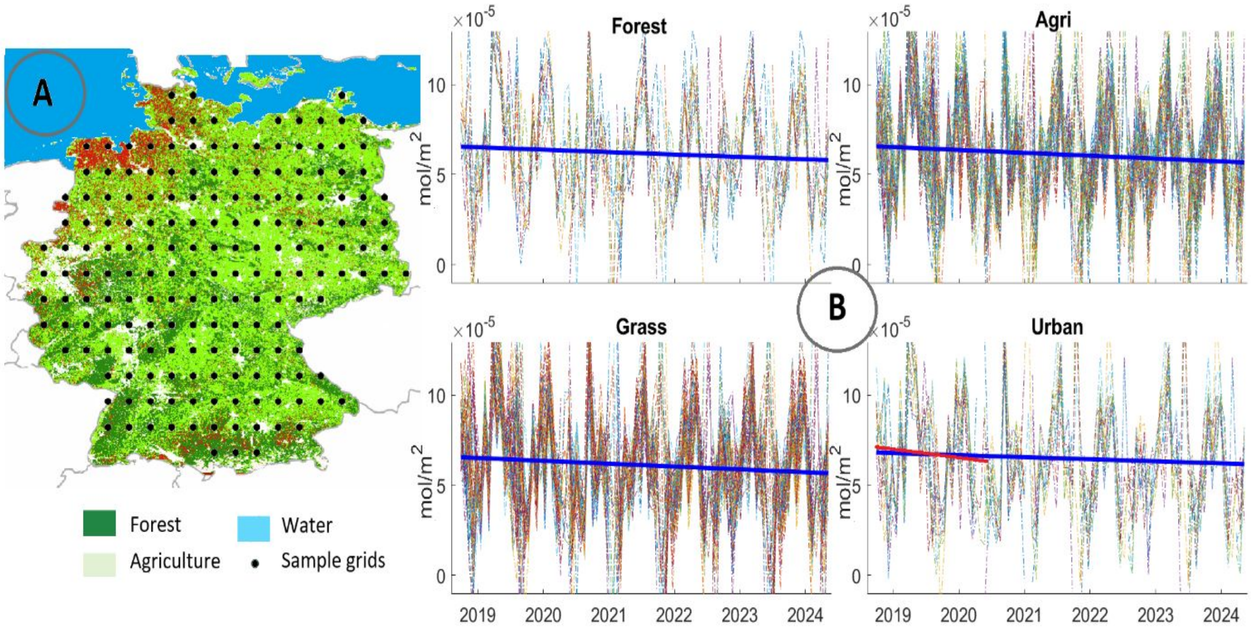

Figure S18. A: sample points over Germany, B: time series of HCHO over four land covers: forest, agriculture, grassland and urban areas.

Although, the concentration of HCHO exhibits spatio-temporal variabilities, influenced by BVOC emission rates, urban and industrial activities, meteorological conditions, and photochemical activity, in regional scales it may serves as a critical marker for BVOC and VOC oxidation processes. The analysis revealed that similar to NDVI trends, agricultural areas exhibit larger decreases in HCHO compared to forests. The urban areas showed a particular pattern in HCHO; In urban areas, HCHO levels are biased about 3% due to the VOCs produced by vehicles (Figure S19, a). Moreover, urban areas exhibit a 23% smaller decrease in HCHO compared to agricultural areas (Figure S19, b), likely due to an increase in vehicle-induced VOCs. Studies suggest an increase in urban air pollution in major cities worldwide, including Berlin, following the temporary decrease observed during the COVID-19 pandemic[30].

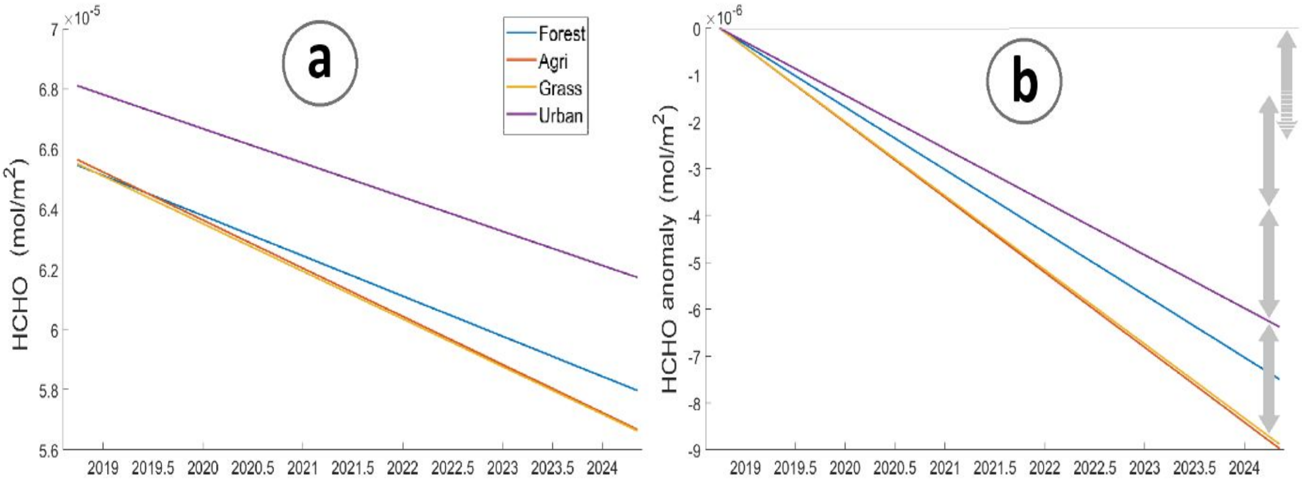

Figure S19. Trends of HCHO: actual quantities (a), and adjusted to zero (b).

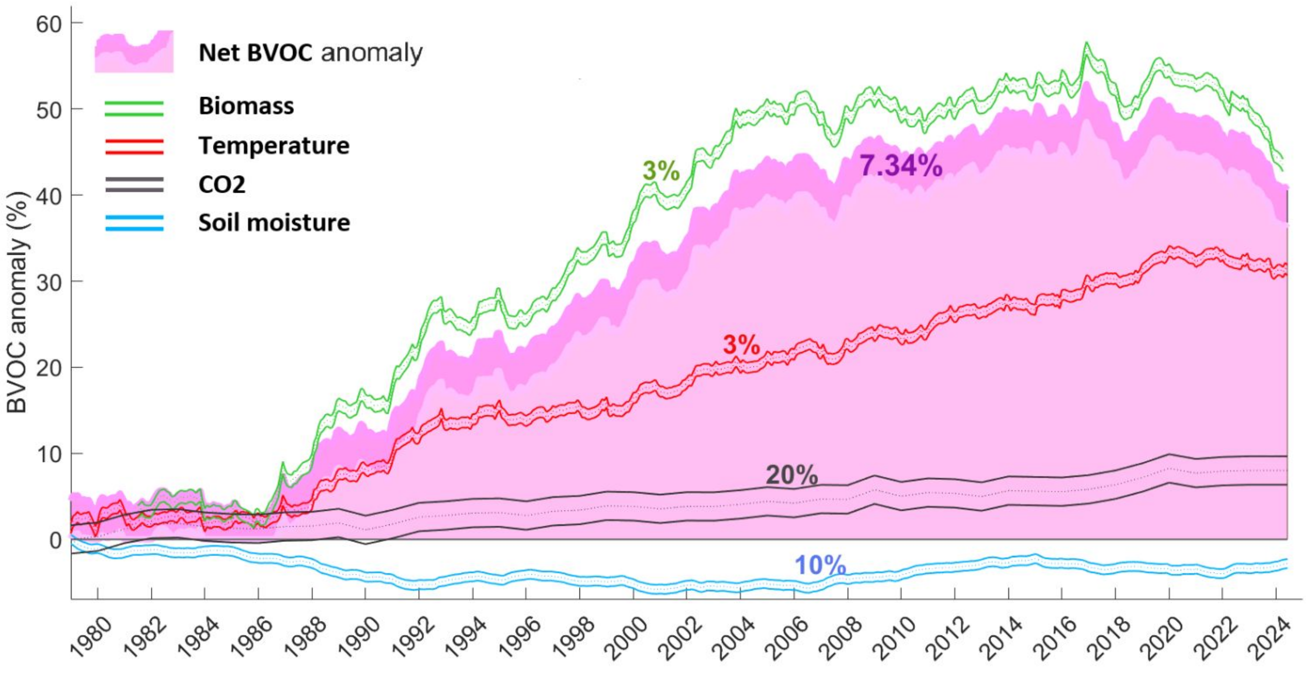

Figure S20. Margin of error for each BVOCs contribution and the resulting accuracy of BVOC anomaly.

References

(1) Destatis- crop statistics: https://www.destatis.de/EN/Themes/Economic-Sectors-Enterprises/Agriculture-Forestry-Fisheries/Field-Crops-Grassland/Tables/arable-land-after-the-main-groups-and-crops.html.

(2) Food and Agriculture Organization of the United Nations. (2020). Global forest resource assessment 2020 report: Germany. FAO. https://www.fao.org/documents/card/en/c/cb0013en/

(3) Bundesministerium für Ernährung und Landwirtschaft (BMEL). (2024). Bundeswaldinventur. Retrieved from [https://www.thuenen.de/en/thuenen-topics/natural-resources-and-protected-goods].

(4) Chudnoff, M. (1984). Tropical timbers of the world (No. 607). US Department of Agriculture, Forest Service.

(5) Forest Products Laboratory (US), 1987. Wood handbook: wood as an engineering material (No. 72). The Laboratory.

(6) Paustian, K., Ravindranath, N. H., & van Amstel, A. R. (2006). 2006 IPCC guidelines for national greenhouse gas inventories.

(7) Kesselmeier, J., & Staudt, M. (1999). Biogenic volatile organic compounds (VOC): an overview on emission, physiology and ecology. Journal of atmospheric chemistry, 33, 23-88.

(8) Hobbs, P. J., Webb, J., Mottram, T. T., Grant, B., & Misselbrook, T. M. (2004). Emissions of volatile organic compounds originating from UK livestock agriculture. Journal of the Science of Food and Agriculture, 84(11), 1414-1420.

(9) Lu, J., Li, S., Liang, G., Wu, X., Zhang, Q., Gao, C., Li, J., Jin, D., Zheng, F., Zhang, M. and Abdelrhman, A.A. (2021). The contribution of microorganisms to soil organic carbon accumulation under fertilization varies among aggregate size classes. Agronomy, 11(11), 2126.

(10) Romero-Olivares, A. L., Davie-Martin, C. L., Kramshøj, M., Rinnan, R., & Frey, S. D. (2022). Soil volatile organic compound emissions in response to soil warming and nitrogen deposition. Elem Sci Anth, 10(1), 00065.

(11) Guenther, A., Hewitt, C.N., Erickson, D., Fall, R., Geron, C., Graedel, T., Harley, P., Klinger, L., Lerdau, M., McKay, W.A. and Pierce, T. (1995). A global model of natural volatile organic compound emissions. Journal of Geophysical Research: Atmospheres, 100(D5), 8873-8892.

(12) Filella, I., Wilkinson, M. J., Llusia, J., Hewitt, C. N., & Peñuelas, J. (2007). Volatile organic compounds emissions in Norway spruce (Picea abies) in response to temperature changes. Physiologia Plantarum, 130(1), 58-66.

(13) Sonwani, S., Saxena, P., Kulshrestha, U. (2016). Role of Global Warming and Plant Signaling in BVOC Emissions. In: Kulshrestha, U., Saxena, P. (eds) Plant Responses to Air Pollution. Springer, Singapore. https://doi.org/10.1007/978-981-10-1201-3_5.

(14) Insam, H., & Seewald, M. S. (2010). Volatile organic compounds (VOCs) in soils. Biology and fertility of soils, 46, 199-213.

(15) Zhang-Turpeinen, H., Kivimäenpää, M., Aaltonen, H., Berninger, F., Köster, E., Köster, K., Menyailo, O., Prokushkin, A. and Pumpanen, J. (2020). Wildfire effects on BVOC emissions from boreal forest floor on permafrost soil in Siberia. Science of the Total Environment, 711, 134851.

(16) Sharkey, T. D., Loreto, F., & Delwiche, C. F. (1991). High carbon dioxide and sun/shade effects on isoprene emission from oak and aspen tree leaves. Plant, Cell & Environment, 14(3), 333-338.

(17) Tognetti, R., Johnson, J. D., Michelozzi, M., & Raschi, A. (1998). Response of foliar metabolism in mature trees of Quercus pubescens and Quercus ilex to long-term elevated CO_2. Environmental and Experimental Botany, 39(3), 233-245.

(18) Rosenstiel, T. N., Potosnak, M. J., Griffin, K. L., Fall, R., & Monson, R. K. (2003). Increased CO_2 uncouples growth from isoprene emission in an agriforest ecosystem. Nature, 421(6920), 256-259.

(19) Heald, C. L., Wilkinson, M. J., Monson, R. K., Alo, C. A., Wang, G., & Guenther, A. (2009). Response of isoprene emission to ambient CO_2 changes and implications for global budgets. Global Change Biology, 15(5), 1127-1140.

(20) Wilkinson, M.J., Monson, R.K., Trahan, N., Lee, S., Brown, E., Jackson, R.B., Polley, H.W., Fay, P.A. and Fall, R.A.Y. Leaf isoprene emission rate as a function of atmospheric CO_2 concentration. Global Change Biology, 15(5), 1189-1200.

(21) Calfapietra, C., Pallozzi, E., Lusini, I., & Velikova, V. (2013). Modification of BVOC emissions by changes in atmospheric [CO_2] and air pollution. Biology, controls and models of tree volatile organic compound emissions, 253-284.

(22) Loreto, F., Fischbach, R. J., Schnitzler, J. P., Ciccioli, P., Brancaleoni, E. N. Z. O., Calfapietra, C., & Seufert, G. (2001). Monoterpene emission and monoterpene synthase activities in the Mediterranean evergreen oak Quercus ilex L. grown at elevated CO_2 concentrations. Global Change Biology, 7(6), 709-717.

(23) Morfopoulos, C., Sperlich, D., Peñuelas, J., Filella, I., Llusià, J., Medlyn, B.E., Niinemets, Ü., Possell, M., Sun, Z. and Prentice, I.C. (2014). A model of plant isoprene emission based on available reducing power captures responses to atmospheric CO 2. New Phytologist, 203(1), pp. 125-139.

(24) Possell, M., & Hewitt, C. N. (2011). Isoprene emissions from plants are mediated by atmospheric CO_2 concentrations. Global Change Biology, 17(4), 1595-1610.

(25) Monson, R. K., Jones, R. T., Rosenstiel, T. N., & Schnitzler, J. P. (2013). Why only some plants emit isoprene. Plant, Cell & Environment, 36(3), 503-516.

(26) Ritchie, H., Roser, M., & Rosado, P. (2020). CO_2 and greenhouse gas emissions. Our world in data.

(27) Gkatzelis, G.I., Coggon, M.M., Stockwell, C.E., Hornbrook, R.S., Allen, H., Apel, E.C., Bela, M.M., Blake, D.R., Bourgeois, I., Brown, S.S. and Campuzano-Jost, P. (2024). Parameterizations of US wildfire and prescribed fire emission ratios and emission factors based on FIREX-AQ aircraft measurements. Atmospheric Chemistry and Physics, 24(2), 929-956.

(28) Mann, M.L., Batllori, E., Moritz, M.A., Waller, E.K., Berck, P., Flint, A.L., Flint, L.E. and Dolfi, E. (2016). Incorporating anthropogenic influences into fire probability models: Effects of human activity and climate change on fire activity in California. PLoS One, 11(4), e0153589.

(29) Stewart, C., Damby, D.E., Horwell, C.J., Elias, T., Ilyinskaya, E., Tomašek, I., Longo, B.M., Schmidt, A., Carlsen, H.K., Mason, E. and Baxter, P.J. (2022). Volcanic air pollution and human health: recent advances and future directions. Bulletin of Volcanology, 84(1), 11.

(30) Sarmadi, M., Rahimi, S., Rezaei, M., Sanaei, D., & Dianatinasab, M. (2021). Air quality index variation before and after the onset of COVID-19 pandemic: a comprehensive study on 87 capital, industrial and polluted cities of the world. Environmental Sciences Europe, 33, 1-17.
